# Inhibitor and substrate cooperate to inhibit amyloid fibril elongation of α-synuclein†

**DOI:** 10.1039/d0sc04051g

**Published:** 2020-09-28

**Authors:** Emil Dandanell Agerschou, Vera Borgmann, Michael M. Wördehoff, Wolfgang Hoyer

**Affiliations:** Institut für Physikalische Biologie, Heinrich-Heine-Universität Düsseldorf 40225 Düsseldorf Germany wolfgang.hoyer@hhu.de; Institute of Biological Information Processing, Structural Biochemistry (IBI-7), JuStruct: Jülich Center for Structural Biology, Forschungszentrum Jülich 52425 Jülich Germany

## Abstract

In amyloid fibril elongation, soluble growth substrate binds to the fibril-end and converts into the fibril conformation. This process is targeted by inhibitors that block fibril-ends. Here, we investigated how the elongation of α-synuclein (αS) fibrils, which are associated with Parkinson's disease and other synucleinopathies, is inhibited by αS variants with a preformed hairpin in the critical N-terminal region comprising residues 36–57. The inhibitory efficiency is strongly dependent on the specific position of the hairpin. We find that the inhibitor and substrate concentration dependencies can be analyzed with models of competitive enzyme inhibition. Remarkably, the growth substrate, *i.e.*, wild-type αS, supports inhibition by stabilizing the elongation-incompetent blocked state. This observation allowed us to create inhibitor–substrate fusions that achieved inhibition at low nanomolar concentration. We conclude that inhibitor–substrate cooperativity can be exploited for the design of fibril growth inhibitors.

## Abstract 2

Amyloid fibril elongation of α-synuclein can be described with the Michaelis–Menten model, where α-synuclein monomer plays a dual role by serving as growth substrate as well as supporting the competitive inhibitor CC48 in blocking fibril ends.

## Graphical abstract

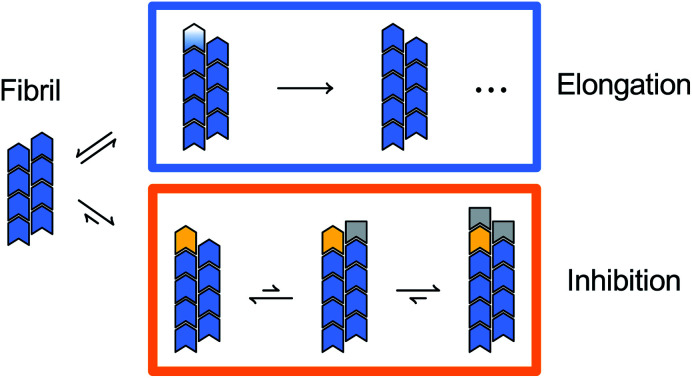

## Introduction

A growing number of proteins have been shown to undergo an autocatalytic aggregation reaction where soluble polypeptide chains convert to insoluble 1D quasi-crystals exhibiting cross-β conformation.^1–3^ When proteins are found in this state they are referred to as amyloid fibrils. The amyloid state is thought to be a generic state that all proteins can adopt and is associated with several diseases, especially neurodegenerative ones.^2–5^

In this paper we focus on the protein α-synuclein (αS) which is believed to play a central role in the pathology of Parkinson's Disease (PD). In PD, αS is found in insoluble inclusions, termed Lewy bodies, where it is thought to predominantly inhabit the amyloid state.^6–9^

Amyloid fibril formation is a multi-step reaction that minimally includes primary nucleation and elongation, but commonly involves additional reactions including secondary nucleation, fragmentation, and competition from so-called off-pathway reactions.^10–13^ This complexity can make interpretation of experimental data exceedingly complicated.^10,14^ However, all of these reaction steps are amenable to modulation by ligands, affording a range of therapeutic opportunities that target different sites on distinct species on the aggregation pathway.^15^ Importantly, molecules that are able to interact with specific sites/species can also provide insight into the mechanism of amyloid formation.^16^

Here we will focus on elongation of fibrils which is the most frequent process in amyloid formation. Elongation of αS fibrils can be studied in isolation using specific solution conditions.^17^ During elongation, a free αS monomer, which in its free state is intrinsically disordered,^18^ (i) absorbs onto the fibril-end and (ii) converts into the specific structure of the templating fibril.^12^ This is reminiscent of enzyme kinetics, and elongation can be treated as a two-step enzymatic reaction, in which fibril-end and monomer serve as catalyst and substrate, respectively.^19–21^

**Fig. 1:**
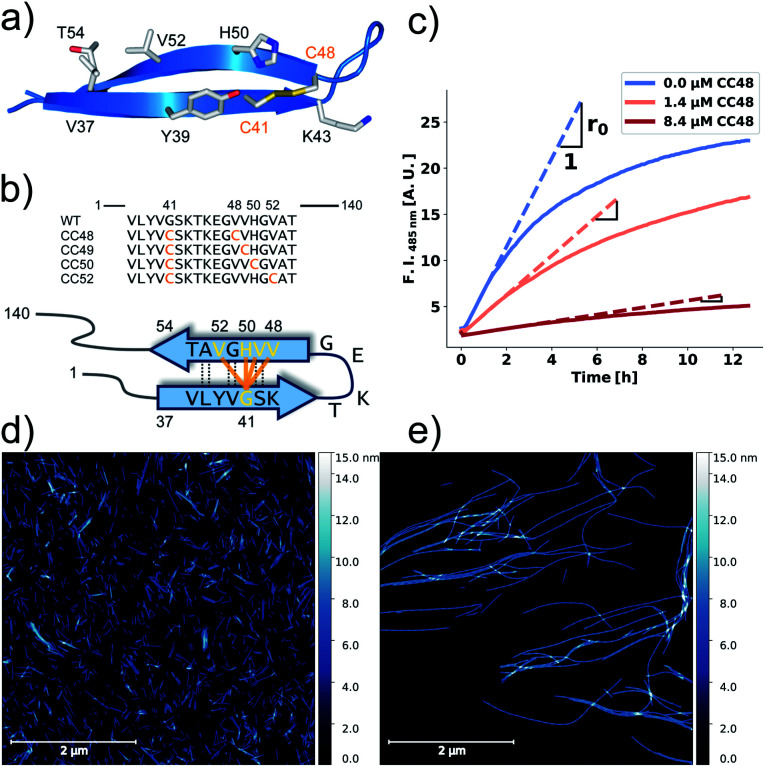
αS variants containing disulfide-stabilized hairpins inhibit elongation of WT αS fibrils. (a) Model of a β-hairpin conformation of CC48 based on the NMR structure of αS bound to β-wrapin AS69 (PDB: 4bxl). (b) Overview of investigated CC mutants, including a scheme of the disulfide bond positions (orange lines) with respect to the β-sheet registry of the hairpin shown in (a). Dashed lines indicate hydrogen bonding across the strands. (c) Exemplary time courses of Tht fluorescence where 25 μM WT monomer was mixed with 10% seeds and allowed to elongate in absence or presence of the inhibitor CC48. The initial slopes, *r*, are extracted by fits to a linear equation (discontinuous lines), where *r*_0_ is the slope when no inhibitor was present. (d and e) AFM imaging of seeds before elongation (d), and after elongation (e) in the presence of 25 μM WT monomer and 0.472 μM CC48–CC48 dimer.

Proteins and peptides have been designed to specifically inhibit the elongation of αS fibrils,^22–25^*e.g.*, by aiming to dock complementary β-strands onto the open β-sheets at the fibril-ends, to prevent the catalytic site from guiding the conformational conversion of further monomers. However, understanding how monomers and inhibitors get incorporated at fibril-ends is still a subject of active research.^12^ We have previously reported that a double cysteine αS mutant containing the amino acid exchanges G41C and V48C, here denoted CC48, inhibits the elongation of wild-type (WT) αS fibrils.^26^ This double exchange introduces an intramolecular disulfide bond that is important for inhibitory activity of CC48. The positions of the two cysteines were chosen to promote the formation of a β-hairpin motif in the region spanning residues 36–57, a region we previously observed to be in complex with an αS monomer-binding protein, the β-wrapin AS69 (Fig. 1a and b).^27,28^

The strong inhibitory effects of both CC48 and AS69 on αS fibril formation highlight the importance of this region, which contains several of the disease associated mutations (E46K, H50Q, G51D, A53E, and A53T).^7,29–32^ This was corroborated recently when an αS deletion mutant lacking residues 36–42 and 45–57 was shown not to aggregate.^33^ In the present work, we investigated sequence requirements and mechanism of the inhibition of αS fibril elongation achieved by CC48 and related constructs. We observe inhibitor–substrate cooperativity, which provides insight into blocked fibril-end states and supports the design of improved inhibitors.

## Results and discussion

### Validation of elongation assay

We performed elongation assays by incubating 2.5 μM preformed and sonicated fibrils (seeds) with WT monomer. Our specific choice of elongation reaction conditions were tested by measuring the rate of elongation using Thioflavin T (Tht) fluorescence over time (Fig. 1c), as Tht is an amyloid specific dye that drastically increases its fluorescence when binding to amyloid fibrils.^34^ As elongation is a bimolecular reaction, the initial rates, *r*, should be directly proportional to available fibril-ends and initial WT αS monomer concentration. The initial rates were extracted by fitting linear curves to the initial slopes as shown in Fig. 1c (see ESI† for the theoretical considerations). To further validate that elongation was the only reaction occurring, atomic force microscopy (AFM) was conducted on seeds before (Fig. 1d) and after (Fig. 1e) elongation. While indeed only short fibrils could be found initially, much longer fibrils were dominating after the Tht time course measurement. Lastly, SDS-PAGE of selected samples from kinetic experiments was performed. The overwhelming amount of protein was found in the insoluble pellet fractions, and the final Tht values correlated with protein amount found in pellet (Fig. S1†). Based on AFM and the non-sigmoidal shape of the Tht time course measurements, it could safely be assumed that elongation was the only amyloid-generating reaction occurring in our setup.

### Disulfide position dependency of inhibition

Our first experimental goal was to gauge the dependency of the inhibitory activity of CC48 on the precise position of the disulfide bond. In addition to CC48, we therefore generated a set of double cysteine αS variants by systematically mutating residues 49 through 52 into cysteines while keeping the other end of the disulfide fixed at position 41 (Fig. 1b). All mutants except CC51, which only resulted in low yield and many impurities, were obtained in monomeric form.

**Fig. 2:**
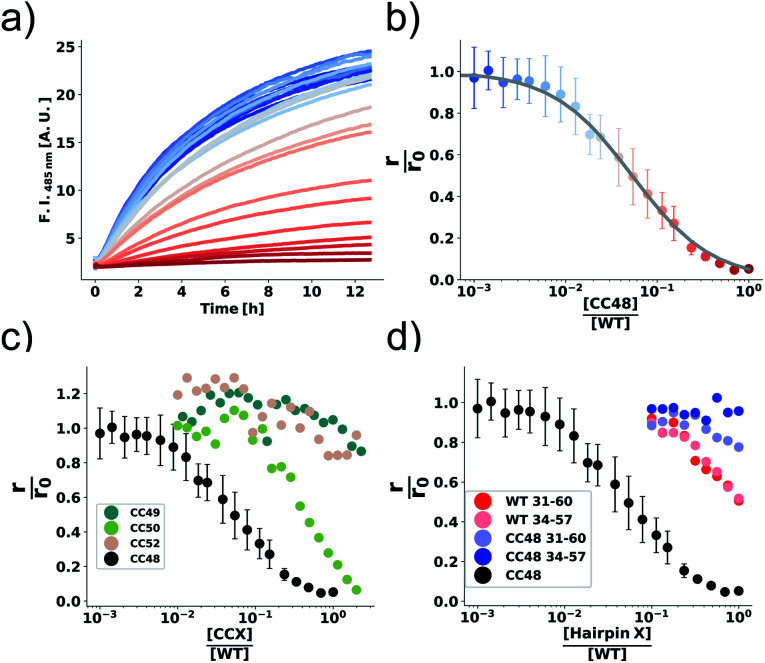
The inhibition efficiency is strongly dependent on the position of the disulfide bond. (a) Elongation experiment performed in the presence of increasing concentrations of CC48 and (b) the mean relative initial slopes, 
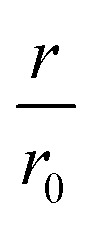
, extracted from four independent experiments. The solid line shows a fit to the competitive inhibitor (FI) model. Error bars correspond to the standard deviations (SD). (c) Relative initial slopes of WT elongation in the presence of the different CC variants and (d) WT and CC48 hairpin peptides. Note that the concentration axis in (b–d) is logarithmic and given as the ratio between the inhibitor and the 25 μM WT monomer that was present. For comparison, the CC48 data is also shown in (c) and (d).

The effect of CC48 on WT elongation was determined in the presence of 25 μM WT monomer and increasing concentrations of CC48 (Fig. 2 and S2†). The initial rates, *r*, were extracted and divided by the initial rate when no CC48 was present *r*_0_ (Fig. 2b). A clear inhibition profile curve could be observed where the relative initial rate, 
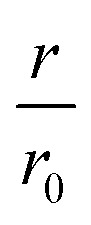
, was halved at a [CC48]/[WT] ratio of 0.054 ± 0.008 ∼ 1/20. It should be noted that CC48 on its own does not elongate WT fibrils unless the elongation is carried out under reducing conditions, here done using the reducing agent dithiothreitol (DTT) (Fig. S7†). This is in line with the incompatibility of the disulfide-induced hairpin with all near-atomic-resolution αS fibril structures reported to date.^35–40^ Adding DTT also severely reduces the inhibitory potential of CC48 (Fig. S8†).

The same type of experiment and data analysis was performed on the newly created CC mutants (Fig. 2c and S3–S5†). Although all mutants were inhibitory to some degree, only CC48 and CC50 inhibited sub-stoichiometrically in terms of the [CCX]/[WT] ratio, indicating specific inhibition, where the latter achieved a halving of 
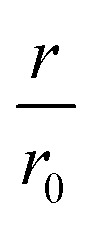
 at a [CC50]/[WT] ratio of 0.43 ± 0.05 ∼ 1/2, an effect that also strongly depended on DTT (Fig. S8†). On the other hand, neither CC52 nor CC49 were particular inhibitory. This position dependency of inhibitory activity is remarkable, especially for the low inhibitory variant CC49 where the variable cysteine is located exactly in between its positions in the highly inhibitory variants CC48 and CC50. Furthermore, the variable exchange is valine to cysteine in CC49, just as in CC48. This argues for a structure-specific origin of the inhibitory activity, perhaps related to formation of a specific β-hairpin.

β-Hairpins are stabilized by cross-strand disulfide bonds between directly opposed cysteine residues at non-hydrogen-bonding positions in the N- and C-terminal β-strands.^41–43^ For the β-hairpin registry shown in Fig. 1b, residues 41 and 50 lie at such directly opposed non-hydrogen-bonding positions n and c. Apart from interactions between n and c, diagonal side chain-side chain interactions especially between residues n and c-2 can also stabilize β-hairpins.^44^ These positions correspond to residues 41 and 48 in the β-hairpin registry in Fig. 1b. Thus, the disulfide bonds in the two variants CC48 and CC50 may promote the formation of a common β-hairpin conformer. The disulfide in CC49, on the other hand, would not support the same β-hairpin as the side chains of cysteines 41 and 49 would lie on opposite faces. Involvement of a β-hairpin conformer according to the registry displayed in Fig. 1b could therefore explain the position dependency of the inhibitory activity. Promotion of an individual peptide β-hairpin through introduction of favourable cross-strand interactions enhances the population of the β-hairpin conformer, but is usually not sufficient to fully stabilize a defined β-hairpin structure.^44,45^ In line with this, CC48 does not form a stable β-hairpin but remains disordered also in the region spanning residues 36–57.^26^ Nevertheless, the disulfide bond will alter the ensemble of β-hairpin conformers that are populated in this region,^46^ with potential consequences for the interaction with fibril-ends and for the inhibition of fibril elongation.

### β-Hairpin peptides

CC48 was by far the strongest inhibitor and was therefore chosen for further mechanistic studies. It was earlier observed that subtly modified fragments of an amyloidogenic protein can be highly inhibitory.^47,48^ CC48, as well as αS, is intrinsically disordered in solution and as such the β-hairpin region is available for potential binding and interfering with fibril-ends.^26^ To test if the observed inhibition could be explained solely by the β-hairpin region of CC48, we performed elongation experiments in the presence of synthetic peptides composed of the β-hairpin region of CC48 as well as the WT sequence (Fig. 2d and S6†). Two different lengths of CC48 β-hairpin peptides were tested, comprising residues 31–60 or 34–57 (pI = 9.14 or 6.74, respectively) and compared to WT peptides (pI = 9.60 or 6.76, respectively). The CC48 hairpin peptides were far less inhibitory than the full-length CC48, meaning that the β-hairpin region alone was not enough to accomplish the observed inhibition. This indicates that (WT) sequence segments beyond the β-hairpin region of CC48 are required for efficient inhibition. As CC48 substoichiometrically inhibits fibril elongation, it most likely acts on fibril-ends,^15^ the sites where WT monomers dock and convert into the fibril structure. While the CC48 β-hairpin region is required for inhibition, WT sequence segments are obviously essential for binding to the fibril-end.

### Dependence of inhibition on WT monomer concentration

**Fig. 3:**
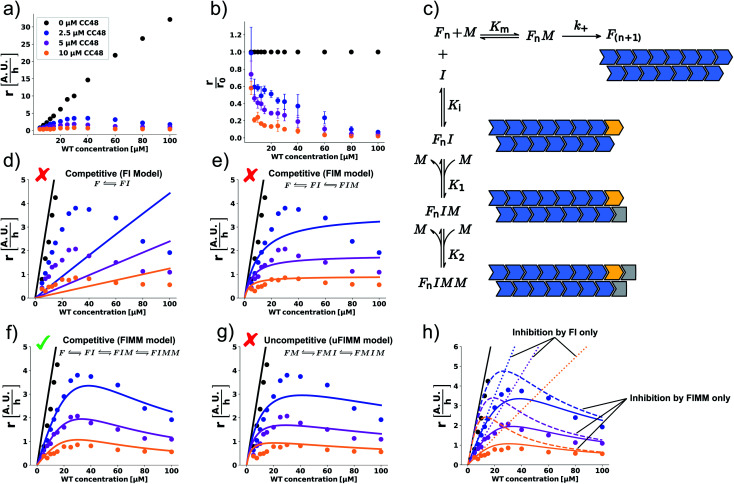
WT monomer cooperates with CC48 in inhibition of WT fibril elongation. (a) WT monomer concentration dependence of the initial slopes, *r*, in the presence of different concentrations of CC48. (b) Mean relative initial slopes, 
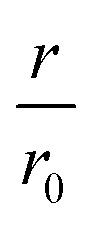
, extracted from three independent experiments, error bars correspond to the SD. (c) Reaction mechanism where the horizontal reaction is elongation and the vertical one describes inhibition. M is a WT monomer, I is a CC48 monomer, and F is a fibril-end. (d–f) Zoom-in of the data shown in (a) fitted (solid lines) to competitive inhibitor models where the inhibitory species are (d) FI, (e) FI and FIM, (f) FI, FIM, and FIMM. (g) Fit to an uncompetitive model where the inhibitor does not bind until a monomer has docked onto the fibril-end, resulting in inhibitory complexes FMI and FMIM. (h) Simulation using the parameters obtained from (f) of how inhibition would appear if only FI or FIMM were inhibitory.

Using ideas from enzymology, which has a long tradition of investigating inhibition mechanisms, we postulate that the mechanism of elongation inhibition by CC48 is analogous to competitive inhibition of enzyme catalysis. Specifically we suggest that CC48 is similar enough to WT monomer to compete for attachment to the fibril-end, where it forms a tight complex, possibly supported by the structural modification in the β-hairpin region. In contrast to WT, however, CC48 bound to the fibril-end cannot serve as a template for incorporation of further monomers to extend the fibril structure. Thus, CC48 suspends the catalytic activity of the fibril-end. The observed inhibition curve was compatible with competitive inhibition with a ∼20-fold higher affinity of CC48 for the WT fibril-end than WT monomer (Fig. 2b). However, the inhibition curve obtained at a varying inhibitor (CC48) concentration and constant substrate (WT) concentration is not sufficient to determine the inhibition mechanism and affinities, as its shape is compatible with a wealth of different mechanisms. When both the substrate and the inhibitor concentrations are varied, a drastic increase in features for identifying the precise mechanism becomes available.^23^ Such experiments revealed a remarkable dependence of the initial rate, *r*, on both the WT and CC48 concentrations (Fig. 3a and S9†). In the absence of CC48, *r* increased almost linearly with WT concentration, in line with fibril elongation by monomer addition to non-saturated fibril-ends. When CC48 was present, *r* initially increased with increasing WT concentration, indicating competitive inhibition (see ESI† theoretical section). But rather than continuing this trend, *r* reached a maximum and began declining. This rather surprising observation indicates that the substrate of the reaction, *i.e.* WT monomer, joined forces with the inhibitor, CC48, to increase the efficacy of the inhibitor.

This WT monomer concentration effect was clearly captured by the relative initial slopes, where a constant decline with respect to the uninhibited sample at the same WT monomer concentration was observed (Fig. 3b). The unusual WT monomer dependency cannot be explained by the standard competitive inhibition model that attributes inhibitory activity only to the complex FI formed from fibril-end (F) and inhibitor CC48 (I) (Fig. 3c and d). The cooperation of WT and CC48 in inhibition suggests that FI can recruit further WT monomer (M), which stabilizes the elongation-incompetent blocked state (Fig. 3c).

A model including the formation of the species FIM can account for a deviation from the linear increase but can still not explain the reduction of *r* with WT monomer concentration (Fig. 3c and e). However, when a second WT monomer can stabilize the blocked state by forming the FIMM species, reduction of *r* with WT monomer concentration can be accounted for (Fig. 3c and f). Global fits to a competitive model including the formation of FIM and FIMM species showed good agreement with the data (Fig. 3f).

In enzyme kinetics, an alternative to competitive inhibition is uncompetitive inhibition, where the inhibitor binds to the enzyme–substrate complex. In inhibition of fibril elongation this would correspond to preferential binding of the inhibitor to a fibril-end with docked but unconverted WT monomer, resulting in the FMI species. If such a species is stabilized by forming the FMIM species with a WT monomer, a reduction of *r* with WT monomer concentration can be achieved. However, a global fit to an uncompetitive model with formation of a FMIM species was not in agreement with the data (Fig. 3g).

Global fits to the competitive FIMM model yielded dissociation constants that followed the order *K*_1_ > *K*_m_ > *K*_i_ > *K*_2_ (Fig. 3c and Table S1†). To gain intuition into the role played by the different inhibiting species, we simulated, using the obtained fitting parameters, how *r* would depend on WT monomer concentration if either FI or FIMM were the only inhibitory species (Fig. 3h). FIM was not considered due to its high dissociation constant, *K*_1_, which results in a negligible population of FIM. According to the simulations, the FI species accounts for the WT monomer concentration dependence at low monomer concentration but does not account for the maximum nor for the decline in elongation rate (Fig. 3h). The FIMM species, on the other hand, does not capture the efficient and CC48 concentration-dependent inhibition at low WT monomer concentrations but accounts for the peak and decline of *r* at high WT monomer concentrations.

According to the obtained equilibrium constants, binding of WT monomer to FIM is much more favourable than to FI (*K*_1_ ≫ *K*_2_). Rationalisation of this observation has to take into consideration that αS fibrils consist of two protofilaments, in which αS subunits are staggered with respect to their neighbours in the other protofilament (schematically depicted in Fig. 3c).^35–40^ Binding of CC48 to the fibril-end might alter the protofilament interface, disfavouring addition of another WT monomer. Once a WT monomer attaches to FI nonetheless, a structurally different binding site with high affinity for an additional WT monomer is created. While the kinetic data does not provide structural information on the different fibril-end complexes, it indicates that at least two WT monomers cooperate with the CC48 inhibitor to form a stabilized blocked state that is incompatible with fibril elongation.

### Inhibition by substrate–inhibitor fusions

**Fig. 4:**
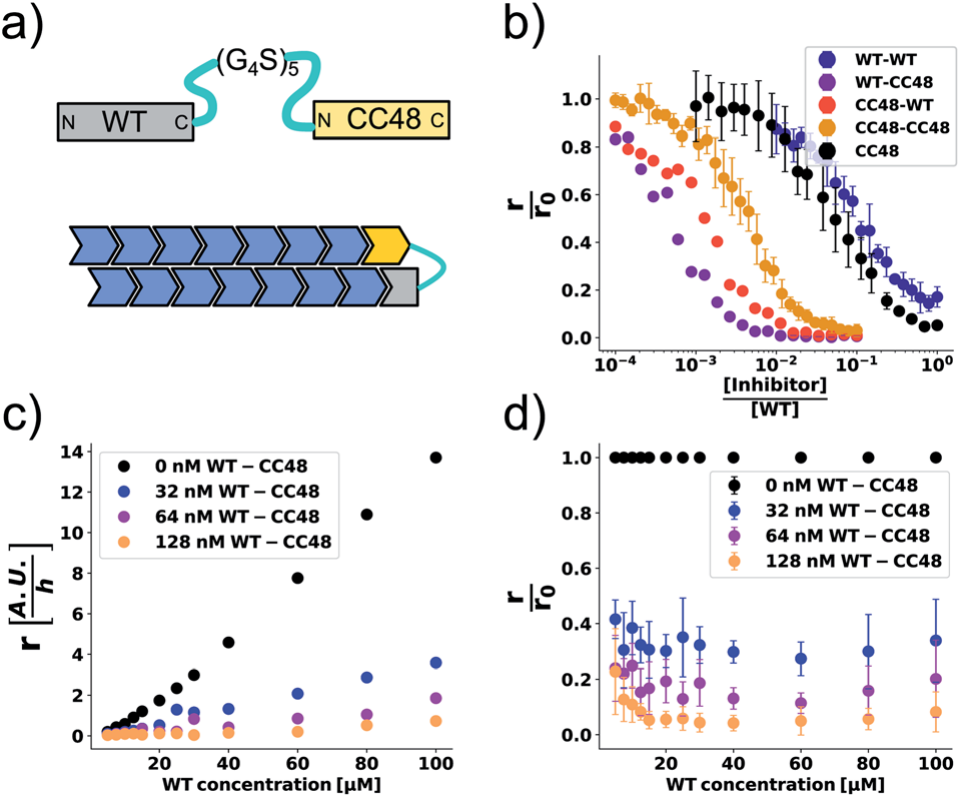
CC48–WT fusion inhibits elongation of WT αS fibrils at low nanomolar concentrations. (a) Schematic overview of the dimer constructs of combinations with zero, one, or two CC48 and WT with a flexible (G_4_S)_5_ linker in between, here exemplified by WT–CC48 dimer. (b) Relative initial rates, 
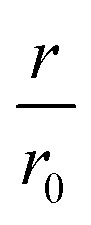
, of elongation assays with increasing concentrations of the dimer constructs at constant WT concentration. The CC48 data is the same as shown in Fig. 2b. (c) WT monomer concentration dependence of the initial slopes, *r*, in presence of different concentrations of WT–CC48. (d) The average 
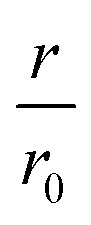
 of the WT–CC48 monomer dependency investigations. Error bars, where present, correspond to the SD.

The cooperation of CC48 with WT monomers in inhibition suggests that an improved inhibitor could be designed by combining CC48 and WT in fusion constructs. As formation of the FIM complex from FI and M was the least favoured step on the inhibition path, IM fusion constructs consisting of one CC48 and one WT unit might show increased inhibitory activity by bypassing this step. We recombinantly expressed dimeric constructs of WT and CC48 separated by flexible linkers as shown schematically in Fig. 4a.

In addition to two heterodimeric constructs WT–CC48 and CC48–WT that differ by the order of WT and CC48 with respect to the linker, we also constructed two homodimers WT–WT and CC48–CC48. The relative initial rates of elongation assays in the presence of these dimers are shown in Fig. 4b and S13–S16.† In agreement with the design concept, the heterodimeric species were far more inhibitory than CC48 as the concentration needed to achieve half relative initial rate corresponded to an [inhibitor]/[WT] ratio of 0.00048 ± 0.00005 ∼ 1/2000, *i.e.*, two orders of magnitude less than what was needed for CC48 alone. The heterodimeric constructs were also more inhibitory than the homodimeric ones, showing that it is in fact the particular combination of CC48 and WT that blocks fibril elongation most efficiently. The WT–WT dimer was almost as inhibitory as CC48 alone, a result that is in agreement with what has been observed for similar constructs.^24,49^ The CC48–CC48 dimer also exhibited strongly increased inhibition compared to CC48, which could be an avidity effect. As expected, the heterodimeric construct exhibited much less monomer dependency than what was observed for CC48 alone (Fig. 4c, d and S18–S21†). This is in line with a notion of FIM being the least favoured species on the inhibition path, whose formation is promoted as the inhibitor, *i.e.* CC48, now carries its own co-inhibitor, *i.e.* the WT, in the heterodimeric fusion constructs.

At a WT monomer concentration of 25 μM, the WT–CC48 fusion showed an IC_50_ of 11 ± 1 nM. This compares favourably to previously reported elongation inhibitors based on αS fusions. These inhibitors were based on different design principles, namely transport of steric bulk to the fibril-end or direct linkage of two αS subunits at different positions within the αS sequence, and reached IC_50_ values of 300 nM,^23,50^ or 22 nM.^24^ In one of these approaches, the function of a fused WT monomer is to serve as a fibril-end-binding domain that brings the fused inhibitor domain close to the second protofilament, with the inhibitor acting as steric bulk that impedes incorporation of further WT monomers.^23,50^ While this approach is related to the current study with regard to the fusion of a WT monomer domain to an inhibitor domain, there are crucial differences: First, CC48 forms an inhibiting FI complex without requiring fusion to a WT monomer. Second, WT monomer, *i.e.*, the unmodified substrate of the elongation reaction, stabilizes the CC48-FI state without requiring fusion to an inhibitor domain. Third, the WT monomer concentration dependency of the steric bulk fusions is different from those of CC48 and the CC48–WT dimers,^23^ indicating a different mechanism of inhibition. Nevertheless, all these approaches show that modified versions of αS can block fibril-ends, with the potency determined by the nature of the fused proteins as well as the type of linkage.

Binding of CC48 to the fibril-end creates a templating-incompetent state with an efficiency that is highly dependent on the specific disulfide fusion (Fig. 2c). Can WT monomer also dock to the fibril-end in such templating-incompetent conformations? Real-time observation by AFM or TIRF microscopy of αS fibril elongation in the presence of WT monomers revealed the existence of long-lived stop states,^51,52^ which were subsequently also reported for several other amyloid proteins.^53–56^ These stop states were suggested to be due to docking of the WT monomer on the fibril-end in a templating-incompetent conformation.^52,54,56^ Thus, the inhibitory efficiency of CC48 might be an enhanced representation of a property that is already inherent to WT monomers. Possibly in a similar vein, certain types of post-translational modified αS might inhibit fibril elongation by establishing templating-incompetent fibril-ends, which could for example explain the inhibitory activity reported for dityrosine-modified αS.^49^

## Conclusions

Exploitation of the principle of self-recognition has proven fruitful for the design of amyloid formation inhibitors.^47,48^ Here, we showed that modification of αS by introduction of a hairpin in a critical N-terminal region results in an inhibitor of fibril elongation, whose efficiency is strongly dependent on the precise position of the hairpin. Our data demonstrates that the efficiency of such fibril-end blocking inhibitors may be dramatically enhanced by linkage to WT monomer, as WT monomer is capable of stabilizing the blocked fibril-end state. As a consequence of the catalytic nature of fibril formation,^21^ we find that inhibition of fibril elongation can be analysed along the lines of enzyme inhibition. However, the specific architecture of the fibril-end can lead to atypical inhibitor properties. We observed here that the substrate of the fibril elongation reaction can contribute to inhibition by stabilizing the enzyme–inhibitor complex.

## Author contributions

E. D. A. and W. H. designed the experiments. E. D. A., V. B., and M. M. W. performed the experiments. E. D. A., V. B., and W. H. analyzed the data. E. D. A. and W. H. wrote the manuscript. All authors commented on the manuscript.

## Conflicts of interest

There are no conflicts to declare.

## Supplementary Material

SC-011-D0SC04051G-s001
