# Supplementary material for: Inhibitor and substrate cooperate to inhibit amyloid fibril elongation of α-synuclein
Source: Chem Sci. 2020 Sep 28;11(41):11331–7. doi: 10.1039/d0sc04051g (PMC8162328; doi:10.1039/d0sc04051g)
Supplement: SC-011-D0SC04051G-s001 [file SC-011-D0SC04051G-s001.pdf]

Electronic Supplementary Material (ESI) for Chemical Science.
This journal is © The Royal Society of Chemistry 2020

Electronic Supplementary Information for Inhibitor and substrate cooperate to inhibit amyloid fibril elongation of α-synuclein

Emil Dandanell Agerschou[1], Vera Borgmann[1],
Michael M. Wördehoff[1], and Wolfgang Hoyer[1,2,*]

[1] Institut für Physikalische Biologie, Heinrich-Heine-Universität Düsseldorf, 40225 Düsseldorf, Germany.

[2] Institute of Biological Information Processing, Structural Biochemistry (IBI-7), and JuStruct: Jülich Center for Structural Biology, Forschungszentrum Jülich, 52425 Jülich, Germany.

[*] To whom correspondence should be addressed:
wolfgang.hoyer@hhu.de

1 Materials and Methods

1.1 α-synuclein variants plasmid preparation

CC48 and CC52 were generated by site directed mutagenesis.[1] To generate the CC49, CC50, WT-CC48, CC48-WT, and WT-WT constructs, the respective DNA sequences were obtained from Life Technologies. CC49 and CC50 were cloned into the pT7-7 vector using NdeI and HindIII restriction sites, whereas the remaining constructs were obtained using AQUA cloning.[2] The sequence for CC48-CC48 already cloned into pT7-7 was obtained from Genscript.

1.2 Preparation and transformation of chemically competent *E. coli* BL21(DE3)

20 ml 2YT medium (PanReac AppliChem ITW reagents, Darmstadt, Germany), with antibiotics if appropriate, was inoculated and grown overnight at 37 °C and 180 RPM. 1 ml of the overnight culture was used to inoculate 100 ml 2YT medium, which was then incubated at conditions identical to the overnight

culture. When the 100 ml culture reached an OD_{600} of 0.4-0.5, the culture was divided into two and centrifuged at 2000 g for 20 min at 4 °C, after which the supernatant was discarded. The cell pellets were carefully resuspended in 10 ml containing 30 mM potassium acetate pH 5.8, 100 mM RbCl, 50 mM $MnCl_2$, 15 (v/v)% glycerol and incubated on ice for 5 min. Cells were centrifuged at 2000 g for 5 min at 4 °C, and the supernatant was discarded. The pellets were carefully resuspended in 1 ml 10 mM MOPS pH 6.5, 75 mM $CaCl_2$, 100 mM RbCl, 15 (v/v)% glycerol before the cells were pooled and incubated on ice for 30 min. Aliqouts of 50 µl were made, flashfrozen in liquid nitrogen, and placed at −80 °C.

1.3 Transformation of chemically competent cells

50 µl chemically competent cells (see above) were thawed on ice, for 10 min before 10–100 ng plasmid was added to the cells, and the sample was mixed by gently flicking the tubes. Sample was incubated on ice for 10 min before the sample was mixed again by gently flicking the tube. The sample was placed at 42 °C for 60 s and then immediately transferred to ice for 2 min. 450 µl SOC medium (2 (w/v)%, tryptone, 0.5 (w/v)% yeast extract, 10 mM NaCl, 2.5 mM KCl, 10 mM $MgCl_2$, 10 mM $MgSO_4$, 20 mM glucose) was added to the sample, that was then placed at 37 °C and 800 RPM for 1 h. 100 µl sample was spread on a LB medium (PanReac AppliChem ITW reagents, Darmstadt, Germany) with 1.5 % agar plate inside a Petri dish.

1.4 Preparation of expression system

pT7-7 plasmids encoding the α-synuclein variants were used to transform chemically competent *E. coli* BL21(DE3) cells, that had previously been transformed with the pNatB vector. The pNatB vector encodes for the NatB complex[3] which acetylates amino termini of eukaryotic proteins having the N-terminal amino acid sequence MD. Transformation of chemically competent cells was performed (see above). Glycerol stocks were prepared of all variants by thoroughly mixing 500 µl of overnight culture with 500 µl 60 % glycerol.

1.5 Protein expression and purification

1.5.1 Expression of α-synuclein variants

Expression was performed similar to a previously established protocol.[4,5] 50 ml autoclaved 2YT medium, supplemented with 100 µg/ml ampicillin and 35 µg/ml chloramphenicol, was inoculated with 20 µl *E. coli* BL21(DE3) glycerol stock. The 50 ml culture was left overnight at 37 °C and 160 RPM. 5 ml of the overnight culture was used to inoculate 0.5 L culture of identical composition as the overnight culture and placed at 37 °C at 110 RPM. Cultures were grown until OD_{600} reached 1 before IPTG was added to a final concentration of 1 mM to induce expression, that was carried out for 4 h without changing incubation

conditions. Cells were pelleted by centrifugation at 5000 g for 20 min at 4 °C after which the supernatant was removed. Cells from 0.5 L were resuspended in 7.5–10 ml MilliQ water before being placed at −20 °C for storage.

1.5.2 Purification of α-synuclein variants

Purification was performed similar to previously established protocols.[4,5] The resuspended cell pellet was thawed at ∼ 50 °C before lysis was performed by sonication using a Sonopuls UW 3200 (Bandelin, Berlin, Germany) sonicator with a MS72 probe for 10 min at 35 % maximal amplitude in pulses of 5 s on, 3 s off. The cell lysate was placed at 99 °C for 5 min with frequent vortexing. The lysate was cleared by centrifugation at 13 600 g for 30 min at 4 °C before transferring the supernatant to a clean 50 ml tube. Precipitation was done by dropwise addition of a 4 M solution of $(NH_4)_2SO_4$ to a final concentration of 1.75 M while gently stirring the sample; stirring was done for an additional 3 min before sample was placed on ice for ∼ 10 min. Pelleting was performed by centrifugation at 13 600 g for 20 min at 4 °C before the supernatant was discarded and the pellet was placed at −20 °C. The pellet was resuspended in 3 ml of 25 mM Tris:Cl pH 8 placed inside a Slide-a-lyzer dialysis cassette with a capacity of 3–12 ml and molecular weight cutoff of 3.5 kDa (Thermo Scientific, Rockford IL, USA) and dialysed against 1.4 L of the same buffer for at least 1.5 h. The dialysed sample was loaded onto a 5 ml HiTrap Q HP anion exhange chromatography column (GE Healthcare, Uppsala, Sweden), connected to an Äkta Purifier (GE Healthcare, Uppsala, Sweden). In case of the dimeric constructs, 10 M Urea in 50 mM Tris:Cl pH 8 was loaded onto the column and incubated for 0.5 h. The bound protein was eluted using a linear gradient of NaCl from 0–500 mM (using a buffer containing 25 mM Tris:Cl pH 8, 0.8 M NaCl) where the protein usually began eluting when the conductivity was in the 30–40 mS/cm range and fractions were collected until the ratio of absorbance at 280 nm and 260 nm reached ∼ 1. The fractions that corresponded to the peak from the anion exchange chromatography was pooled and $(NH_4)_2SO_4$ was added to a final concentration of 1.75 M and the sample was left on ice for 10 min before being centrifuged at 13 600 g for 20 min at 4 °C. The supernatant was discarded and the pellet was resuspended in up to 2.5 ml 20 mM MOPS pH 7.4, 50 mM NaCl depending on the size of the pellet. Size exclusion chromatography of the sample was performed by loading the samples onto a Superdex 75 increase 10/300 or a Hiload Superdex 75 16/600 pg. 1.5 ml centred at an elution volume of 12 ml or 8 ml centred at 58 ml was collected for Superdex 75 increase 10/300 and Hiload Superdex 75 16/600 pg respectively.

1.6 α-synuclein concentration determination

Absorbance in the 340–240 nm range was measured with an UV-VIS Spectrophotometer V-650 (JASCO, Pfungstadt, Germany) on a 1:4 dilution of the samples. Concentration was determined using an extinction coefficient defined by the number tyrosine residues times $1400\,M^{-1}\,cm^{-1}$ at 275 nm (*i.e.* $1400\,M^{-1}\,cm^{-1}$

for the hairpin peptides, $5600\,M^{-1}\,cm^{-1}$ for the monomeric constructs, and $11\,200\,M^{-1}\,cm^{-1}$ for dimeric constructs) subtracting the signal at 320 nm.

1.7 Elongation assays using Tht fluorescence

All experiments were performed in 96-well half area assay plates, non-binding surface, black with clear bottom, polystyrene, (REF 3881), from Corning (Kennebunk ME, USA). Furthermore, the outermost wells were never used for samples. Water was added to the empty wells in the immediate vicinity of samples, and the plate was sealed with sealing tape, clear polyolefin (232701) from Thermo Scientific (Rochester NY, USA). Assays were performed on a FLUOstar Omega or Clariostar (BMG Labtech, Ortenberg, Germany) using 12-point orbital averaging with at diameter of 3 mm using bottom optics and a settling time of 1.0 s between measuring each well. Measurements were done every 100 s for the first 100 cycles, subsequent measurements were done every 360 s. For the FLUOstar Omega, a Thioflavin T filter with excitation at 448 nm (10 nm bandwidth) and emission at 482 nm (10 nm bandwidth) was used, and a gain of 800 employed. For the Clariostar, the monochromator was set to excitation at 440 nm (15 nm bandwidth) and emission at 485 nm (20 nm bandwidth), and a gain of 750 was employed.

All assays were performed using 25 µM Thioflavin T UltraPure Grade (Anaspec Inc., Fremont CA, USA) and the reaction buffer was 20 mM MOPS, 50 mM NaCl pH 7.4, and 0.04 % NaN_3. Unless otherwise stated, the samples were prepared at room temperature without seeds to a volume of 90 µl and placed onto the plate. The plate was then sealed and placed at 37 °C inside the plate reader for 10–20 min for temperature equilibration. The seal was removed and seeds injected by manually pipetting 10 µl seed solution (see below) into the relevant wells. The final seed concentration was then 2.5 µM in monomer equivalents. The plate was resealed placed in the plate reader again and measurements carried out. The injection procedure took less than five minutes.

1.7.1 Seed preparation

Seeds were prepared in the same reaction buffer as in Tht fluorescences experiments (see above). Seeds were prepared either by incubating 25 µM WT monomer in a 1.5 ml Eppendorf tube at 37 °C with an added glass bead of 2.85–3.45 mm at 800 RPM for at least 3 days. Alternatively, fibrils from previous elongation experiments were diluted to 25 µM in monomer equivalents. The fibrils were sonicated twice using a UP200St sonicater with a VialTweeter (Hielscher Ultrasound Technology, Teltow, Germany) at 20 s at 70 % maximum amplitude. The VialTweeter did not lead to a high degree of reproducibility of the absolute rates even when fibrils were prepared identically (data not shown), however, it has the advantage of working with closed and sealed tubes, thereby minimising formation of aerosols.

1.8 Gel electrophoresis of monomer and fibrils

Samples of 80 µl were spun at 16 100 g for 30 min using a tabletop centrifuge. 70 µl was removed without disturbing the pellet and the remaining 10 µl was mixed with 70 µl 9 M urea to dissolve the pellet. The pellet samples were then diluted fivefold by additional 9 M urea. 10 µl sample (diluted pellet or undiluted supernatant) was mixed with reducing SDS loading buffer (2 mM dithiothreitol, 2 % SDS, 8 % glycerol, 0.05 % bromophenol blue, 0.05 M Tris:Cl pH 6.8), and incubated for 1 h at room temperature. Samples were loaded onto a Tris/glycine/SDS buffer (Bio-Rad, Feldkirchen, Germany) 15 % polyacrylamide gel made from Rothiphorese 30 (37.5:1) (Carl Roth) and cast on. Gel electrophoresis was performed on a Mini-PROTEAN Tetra System (Bio-Rad, Feldkirchen, Germany), and the gels were stained using SERVA Blue R (equivalent to Coomassie brilliant blue R-250) (SERVA, Heidelberg, Germany). Transilluminated images of the gels were recorded on a ChemiDoc MP* (Bio-Rad, Feldkirchen, Germany), using the standard coomassie filter. The images were exported as 16-bit images (export for analysis feature), lanes were marked and plotted using the Gel Analyzer tool from ImageJ, and the baseline subtracted area of the peaks corresponding to $\sim$ 15 kDa bands were extract from each lane excluding the marker lane.

1.9 Atomic force microscopy (AFM)

Endpoint samples of several kinetic experiments where inhibitor dependency was investigated were prepared for AFM by first transferring the entire content of the wells into clean 1.5 ml Eppendorf tubes. From these tubes, 10 µl (25 µM in WT monomer equivalents in reaction buffer) was placed on freshly cleaved mica and incubated for 10–20 min. Samples were then washed by careful addition of 100 µl dH_2O that was immediately removed, a procedure that was repeated five times. Samples were dried under a gentle stream of nitrogen. AFM was performed on a Bruker Mulitmode 8 (Billerica, Massachusetts, USA) using ScanAsyst-Air cantilevers (Camarillo, California, USA) using the ScanAsyst PeakForce tapping mode in air. The data was imported into Gwyddion and background correction was performed by aligning rows using the median.

2 Modelling of the mechanisms of inhibition

In order to understand which chemical mechanism would be able to explain the observed inhibition of CC mutants, we tested different models of amyloid growth. Regardless of the exact inhibition model, it was assumed that:

- The total concentration of fibril-ends does not change during the initial elongation (or indeed throughout the growth reaction).
- The total concentration of fibril ends is much smaller than WT monomer concentration.

- The free WT monomer concentration remains constant and equal to the total monomer concentration during the initial elongation.
- The free inhibitor concentration remains constant and equal to the total inhibitor concentration during the initial elongation.

What follows is a description of how the models of growth and inhibition can be derived and which assumptions are used in doing so.

2.1 Competitive inhibition (FI) model

Tentative similarities between fibril elongation kinetics and enzyme kinetics have previously been suggested. In particular, non-linear dependency of the elongation rate at the high concentration monomer regime have been observed for several amyloidogenic proteins including α-synuclein and dock-lock models have been proposed for others.[6–11] As noted earlier, this could potentially be explained by a Michaelis–Menten-like mechanism. Although linearity in monomer concentration was observed here (in the absence of inhibitor), the underlying elongation mechanism could still be susceptible to Michaelis–Menten inhibition types known from enzyme kinetics. The CC mutants are almost identical to monomer, they could still bind to the fibril-end, but, due to their alteration in a region that is commonly found in the fibril core,[12–17] not be able to convert into a fibrillar conformation. This would block the end from growth until dissociation occurred. Such a mechanism would be equivalent to the Competitive Inhibition (CI) model from enzyme kinetics, more precisely:

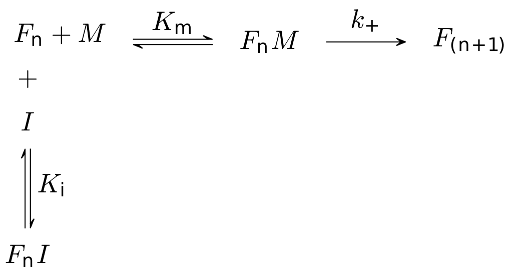

F_n denotes a fibril-end of length n. however, it is assumed that all ends are equivalent, therefore the length will be omitted in the model derivations. M is free WT monomer, F_nM is the complex of a WT monomer bound to a fibril-end but not yet incorporated, K_m is the dissociation constant of WT monomer binding, k_+ is the reaction rate whereby a bound monomer gets incorporated, I is free inhibitor, FI is the unproductive complex between inhibitor and fibril-end, and K_i is the dissociation constant of inhibitor complex formation. Following the formalism from[18], the observed rate of fibril growth is given by conversion of F_nM:

$$r = k_+[FM] \tag{2.1}$$

It is assumed that both complexes are at equilibrium with dissociations constants given by:

$$K_m = \frac{[F][M]}{[FM]} \tag{2.2a}$$

$$K_i = \frac{[F][I]}{[FI]} \tag{2.2b}$$

Conservation of mass for the fibril-end is given by:

$$[F]_T = [F] + [FM] + [FI] \tag{2.3}$$

Deriving the observed elongation rate as function of total monomer, inhibitor, and fibril-end concentration is:

$$\begin{aligned}
\frac{r}{[F]_T} &= \frac{k_+[FM]}{[F] + [FM] + [FI]} \\
&= \frac{k_+\frac{[F][M]}{K_m}}{[F] + \frac{[F][M]}{K_m} + \frac{[F][I]}{K_i}} \\
r &= \frac{R_{\max}[M]}{[M] + K_m\left(1 + \frac{[I]}{K_i}\right)}, \quad R_{\max} = k_+[F]_T
\end{aligned} \tag{2.4}$$

As it is possible to achieve the same total fibril-end concentration within one experiment but difficult to reproduce between different experiments, internal controls where no inhibitor is present can be used to normalise the data.

$$\begin{aligned}
\frac{r}{r_0} &= \frac{\frac{R_{\max}[M]}{[M]+K_m\left(1+\frac{[I]}{K_i}\right)}}{\frac{R_{\max}[M]}{K_m+[M]}} \\
&= \frac{K_m + [M]}{[M] + K_m\left(1 + \frac{[I]}{K_i}\right)}
\end{aligned} \tag{2.5}$$

This model is enough to capture the decreasing relative rate when keeping the WT monomer concentration constant and increasing the inhibitor concentration. In order to understand if it can explain the monomer dependency as well, the limits of Equation 2.5 with respect to WT monomer concentration can be investigated:

$$\frac{r}{r_0} \to 1 \text{ as } [M] \to \infty \tag{2.6a}$$

$$\frac{r}{r_0} \to \frac{1}{1 + \frac{[I]}{K_I}} \text{ as } [M] \to 0 \tag{2.6b}$$

From this it is clear that Equation 2.5 cannot explain the WT monomer dependency as Equation 2.5 would approach 1 at high WT monomer concentration, and not what is observed namely a monotonic decrease in relative rate with increasing WT monomer concentrations.

2.2 Cooperative inhibition (FIM) model

In order to be able to explain the increase in efficiency of the inhibitor as a function of monomer concentration, we modified the model from above by introducing a species where inhibitor, WT monomer, and fibril-end form a ternary inhibitory complex:

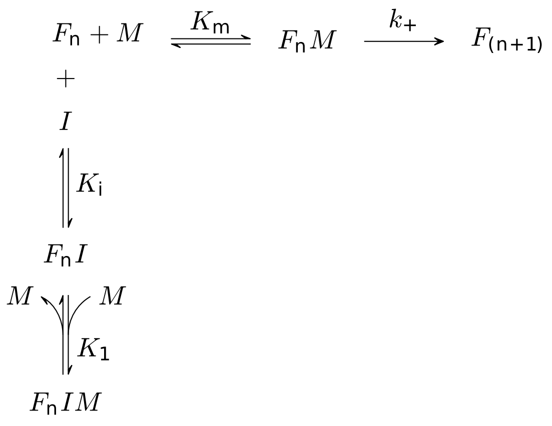

It is assumed that all species are at equilibrium and the dissociation constants and concentrations are given by:

$$K_m = \frac{[F][M]}{[FM]} \tag{2.7a}$$

$$K_i = \frac{[F][I]}{[FI]} \tag{2.7b}$$

$$K_1 = \frac{[FI][M]}{[FIM]} = \frac{[F][I][M]}{K_i[FIM]} \tag{2.7c}$$

Conservation of mass of the fibril-ends now reads:

$$[F]_T = [F] + [FM] + [FI] + [FIM] \tag{2.8}$$

As before, the growth rate is proportional $[FM]$:

$$r = k_+[FM] \tag{2.9}$$

Dividing r by the total concentration of fibril-ends and performing substitutions using Equation 2.8, Equation 2.7:

$$\begin{aligned}
\frac{r}{[F]_T} &= \frac{k_+[FM]}{[F]+[FM]+[FI]+[FIM]} \\
&= \frac{k_+\frac{[F][M]}{K_m}}{[F]+\frac{[F][M]}{K_m}+\frac{[F][I]}{K_i}+\frac{[F][I][M]}{K_iK_1}} \\
r &= \frac{R_{\max}[M]}{K_m\left(1+\frac{[I]}{K_i}\right)+[M]\left(1+\frac{[I]K_m}{K_iK_1}\right)}, \quad R_{\max}=k_+[F]_T \qquad (2.10)
\end{aligned}$$

Again, normalising to total fibril-end concentration:

$$\begin{aligned}
\frac{r}{r_0} &= \frac{\frac{R_{\max}[M]}{K_m\left(1+\frac{[I]}{K_i}\right)+[M]\left(1+\frac{[I]K_m}{K_iK_1}\right)}}{\frac{R_{\max}[M]}{K_m+[M]}} \\
&= \frac{K_m+[M]}{K_m\left(1+\frac{[I]}{K_i}\right)+[M]\left(1+\frac{[I]K_m}{K_iK_1}\right)} \qquad (2.11)
\end{aligned}$$

Which has the limiting behaviour with respect to WT monomer concentration:

$$\frac{r}{r_0} \to \frac{1}{1+\frac{[I]K_m}{K_iK_1}} \text{ as } [M]\to\infty \qquad (2.12a)$$

$$\frac{r}{r_0} \to \frac{1}{1+\frac{[I]}{K_i}} \text{ as } [M]\to 0 \qquad (2.12b)$$

Which, contrary to Equation 2.5, is capable of capturing a decrease in relative rate with increasing WT monomer if $K_m > K_1$. This is however not the only requirement that needs to be satisfied. The non-relative (raw) rate first increases and then decreases at increasing WT monomer concentration in presence of inhibitor. Equation 2.10 is a monotonically increasing function with respect to monomer and therefore cannot capture this aspect.

2.3 Three-step inhibition (FIMM) model

In order to explain the increase in rate with WT monomer concentration followed by a decrease, we extended the FIM model by introducing an additional inhibitory species formed by binding of an additional WT monomer to the ternary (FIM) complex:

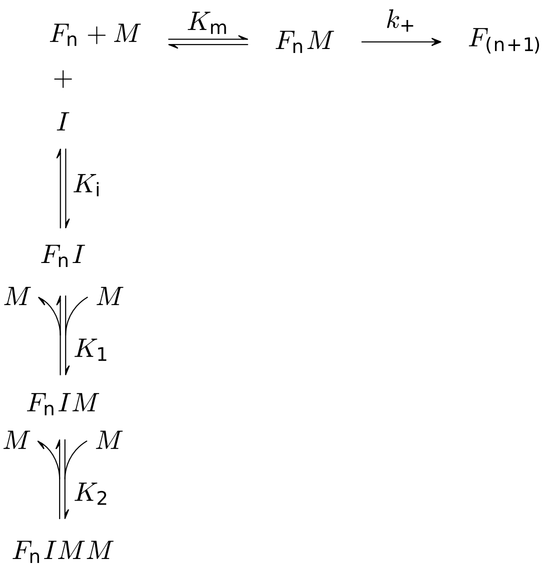

It is assumed that the binary $[FI]$, ternary $[FIM]$, as well as the quaternary complex $[FIMM]$ have reached equilibrium and the dissociation constants, K_1 and K_2 can be defined:

$$K_m = \frac{[F][M]}{[FM]} \tag{2.13a}$$

$$K_i = \frac{[F][I]}{[FI]} \tag{2.13b}$$

$$K_1 = \frac{[FI][M]}{[FIM]} = \frac{[F][I][M]}{K_i[FIM]} \tag{2.13c}$$

$$K_2 = \frac{[FIM][M]}{[FIMM]} = \frac{[F][I][M][M]}{K_i K_1 [FIMM]} \tag{2.13d}$$

Conservation of mass of the fibril-ends now reads:

$$[F]_T = [F] + [FM] + [FI] + [FIM] + [FIMM] \tag{2.14}$$

As before, the growth rate is proportional to $[FM]$:

$$r = k_+[FM] \tag{2.15}$$

Dividing r by the total concentration of fibril-ends and performing substitutions using Equation 2.14 and Equation 2.13:

$$\frac{r}{[F]_T} = \frac{k_+[FM]}{[F] + [FM] + [FI] + [FIM] + [FIMM]}$$

$$= \frac{k_+ \frac{[F][M]}{K_m}}{[F] + \frac{[F][M]}{K_m} + \frac{[F][I]}{K_i} + \frac{[F][I][M]}{K_i K_1} + \frac{[F][I][M][M]}{K_i K_1 K_2}}$$

$$r = \frac{R_{\max}[M]}{K_m\left(1 + \frac{[I]}{K_i}\right) + [M]\left(1 + \frac{[I]K_m}{K_i K_1} + [M]\frac{[I]K_m}{K_i K_1 K_2}\right)} \tag{2.16}$$

Again, normalising to total fibril-end concentration:

$$\frac{r}{r_0} = \frac{\frac{R_{\max}[M]}{K_m\left(1 + \frac{[I]}{K_i}\right) + [M]\left(1 + \frac{[I]K_m}{K_i K_1} + [M]\frac{[I]K_m}{K_i K_1 K_2}\right)}}{\frac{R_{\max}[M]}{K_m + [M]}}$$

$$= \frac{K_m + [M]}{K_m\left(1 + \frac{[I]}{K_i}\right) + [M]\left(1 + \frac{[I]K_m}{K_i K_1} + [M]\frac{[I]K_m}{K_i K_1 K_2}\right)} \tag{2.17}$$

The limiting behaviour is given by:

$$\frac{r}{r_0} \to 0 \text{ as } [M] \to \infty \tag{2.18}$$

$$\frac{r}{r_0} \to \frac{1}{1 + \frac{[I]}{K_i}} \text{ as } [M] \to 0 \tag{2.19}$$

This limiting relative rate behaviour is similar to the FIM model (see Equation 2.12, and therefore is also able to describe the WT monomer dependency of inhibition. Additionally the growth rate given by the FIMM model (see Equation 2.16), contrary to the corresponding one in the FIM model(see Equation 2.10) potentially have extrema, which, if they exists, can be found where $\frac{\partial r}{\partial m} = r'([M]) = 0$ for constant $[I]$. In order to do this, the following substitutions are made:

$$x = [M] \qquad a = R_{\max} \qquad b = K_m\left(1 + \frac{[I]}{[K_i]}\right)$$

$$c = \frac{[I]K_m}{K_i K_1} \qquad d = \frac{[I]K_m}{K_i K_1 K_2}$$

$$g = ax \qquad h = b + x + xc + dx^2 \qquad r([M]) = y \tag{2.20}$$

Which leads to:

$$
\begin{aligned}
y &= \frac{g}{h} \\
y' &= \frac{g'h - gh'}{h^2} \\
y' &= \frac{a\left(b + x + cx + dx^2\right) - ax\left(1 + c + 2dx\right)}{h^2} \\
y' &= \frac{ab - adx^2}{h^2} \\
0 &= ab - adx^2 \\
x &= \pm\sqrt{\frac{b}{d}} \qquad (2.21)
\end{aligned}
$$

This means that there exists an extremum in the positive WT monomer concentration range, which is located at:

$$
\begin{aligned}
[M]_{\max} &= \sqrt{\frac{K_m\left(1 + \frac{[I]}{[K_i]}\right)}{\frac{[I]K_m}{K_i K_1 K_2}}} \\
&= \sqrt{K_1 K_2 \left(\frac{K_i}{[I]} + 1\right)} \qquad (2.22)
\end{aligned}
$$

We're only interested in the positive solutions as x represents concentrations.

2.4 Three-step uncompetitive inhibition (uFIMM) model

An alternative to the FIMM model that has somewhat similar qualitative behaviour is an uncompetitive model where the inhibitor does not bind to the fibril-end before a fibril-end monomer complex has formed:

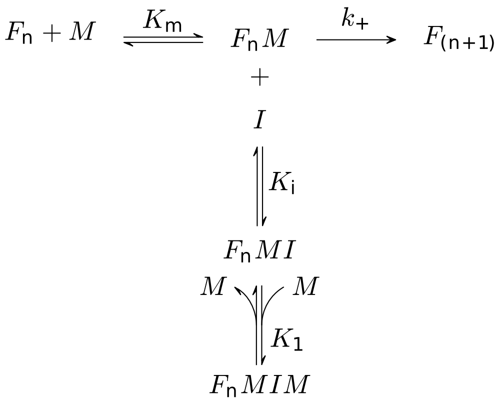

It is assumed that the binary $[FI]$, ternary $[FMI]$, as well the quaternary complex $[FMIM]$ have reached equilibrium and the dissociations K_i and K_1 can be defined:

$$
\begin{aligned}
K_m &= \frac{[F][M]}{[FM]} && (2.23a)\\
K_i &= \frac{[FM][I]}{[FMI]} = \frac{[F][M][I]}{K_m[FMI]} && (2.23b)\\
K_1 &= \frac{[FMI][M]}{[FMIM]} = \frac{[F][M][I][M]}{K_i K_m[FMIM]} && (2.23c)
\end{aligned}
$$

Conservation of mass of the fibril-ends now reads:

$$[F]_T = [F] + [FM] + [FMI] + [FMIM] \tag{2.24}$$

As before, the growth rate is proportional $[FM]$:

$$r = k_+[FM] \tag{2.25}$$

Dividing r by the total concentration of fibril-ends and performing substitutions using Equation 2.24 and Equation 2.23:

$$
\begin{aligned}
\frac{r}{[F]_T} &= \frac{k_+[FM]}{[F] + [FM] + [FMI] + [FMIM]}\\
&= \frac{k_+\frac{[F][M]}{K_m}}{[F] + \frac{[F][M]}{K_m} + \frac{[F][M][I]}{K_m K_i} + \frac{[F][I][M]^2}{K_m K_i K_1}}\\
&= \frac{R_{\max}[M]}{K_m + [M]\left(1 + \frac{[I]}{K_i}\left(1 + \frac{[M]}{K_1}\right)\right)}
\end{aligned}
\tag{2.26}
$$

Normalising to total fibril-end concentration:

$$
\begin{aligned}
\frac{r}{r_0} &= \frac{\dfrac{R_{\max}[M]}{K_m + [M]\left(1 + \frac{[I]}{K_i}\left(1 + \frac{[M]}{K_1}\right)\right)}}{\frac{R_{\max}[M]}{K_m+[M]}}\\
&= \frac{K_m + [M]}{K_m + [M]\left(1 + \frac{[I]}{K_i}\left(1 + \frac{[M]}{K_1}\right)\right)}
\end{aligned}
\tag{2.27}
$$

The limiting behaviour is given by:

$$
\begin{aligned}
\frac{r}{r_0} &\to 0 \text{ as } [M] \to \infty && (2.28)\\
\frac{r}{r_0} &\to 1 \text{ as } [M] \to 0 && (2.29)
\end{aligned}
$$

3 Supplementary figures and data

(a)

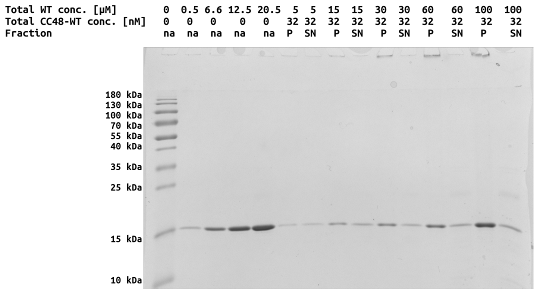

(b)

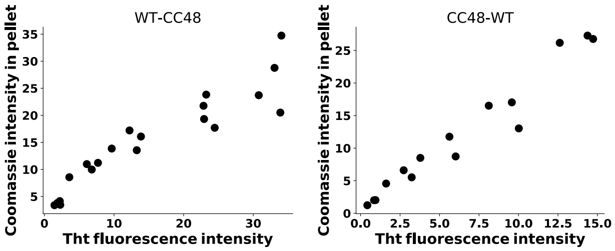

(c)

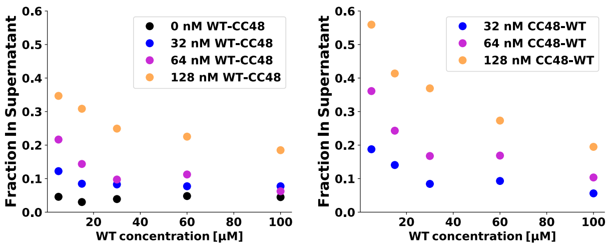

Fig. S1: Validation of elongation conditions. (a) Example of a stained SDS-PAGE gel after a WT monomer dependency experiment in the presence of 32 nM CC48-WT. P and SN indicate pellet and supernatant fractions, respectively. Note that P was diluted five times with respect to the SN (see Materials and Methods above). (b) Correlation between band intensities corresponding to the pellet fractions of the coomassie stained gel vs. the average of the last ten measured points of the Tht fluorescence time traces shown in the last rows of Figure S18 and Figure S20 respectively. Note that the correlation is better at lower Tht signal/monomer concentrations. (c) The fraction $\frac{SN}{SN+5P}$,*i.e.*, monomer left in solution after aggregation of different concentrations of inhibitor and WT monomer.

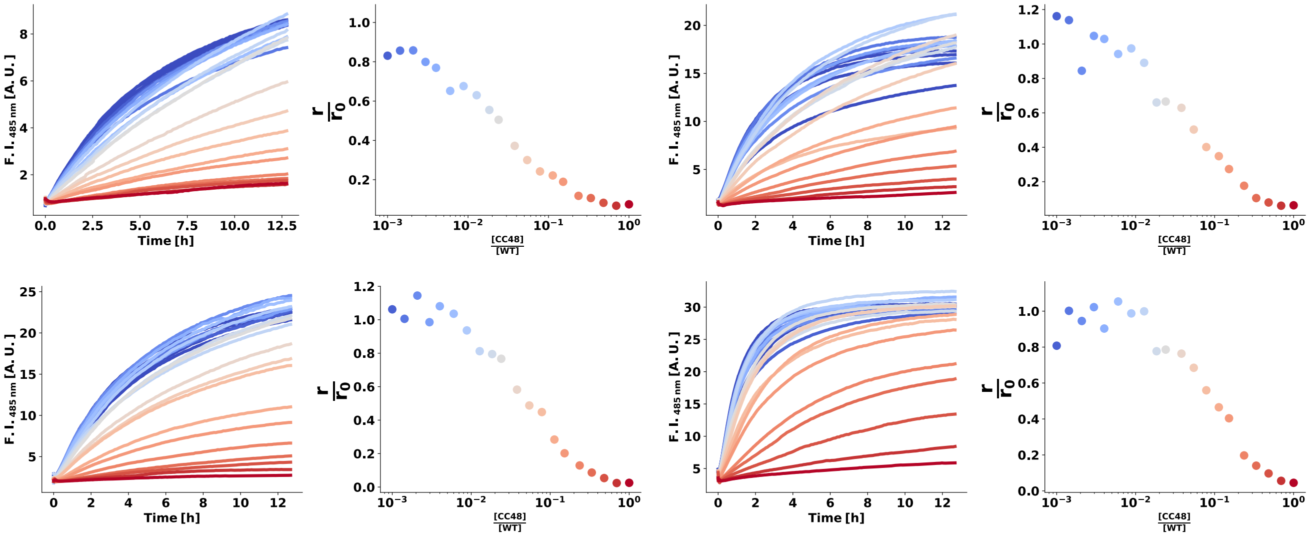

Fig. S2: CC48 dependency of WT fibril elongation. Four independent experiments of Tht fluorescence over time of 25 µM WT monomer, 2.5 µM fibril seeds, with varying concentration of CC48, as well as the extracted relative elongation rates. Concentrations are plotted on a logarithmic x-axis, hence the 0 µM samples are not shown.

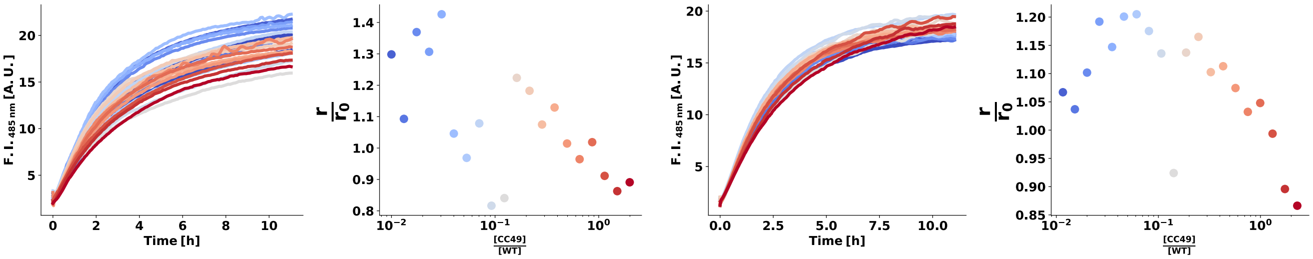

Fig. S3: CC49 dependency of WT fibril elongation. Two independent experiments of Tht fluorescence over time of 25 µM WT monomer, 2.5 µM fibril seeds, with varying concentration of CC49, as well as the extracted relative elongation rates. Concentrations are plotted on a logarithmic x-axis, hence the 0 µM samples are not shown.

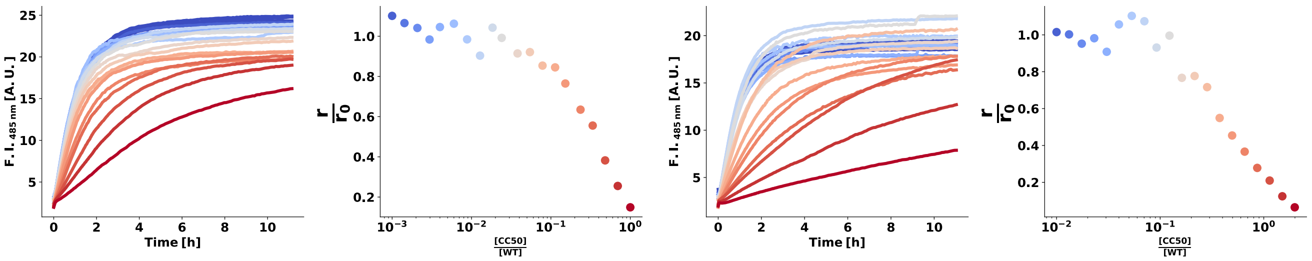

Fig. S4: CC50 dependency of WT fibril elongation. Two independent experiments of Tht fluorescence over time of 25 µM WT monomer, 2.5 µM fibril seeds, with varying concentration of CC50, as well as the extracted relative elongation rates. Concentrations are plotted on a logarithmic x-axis, hence the 0 µM samples are not shown. Note that different concentrations of CC50 was used in the two repeats.

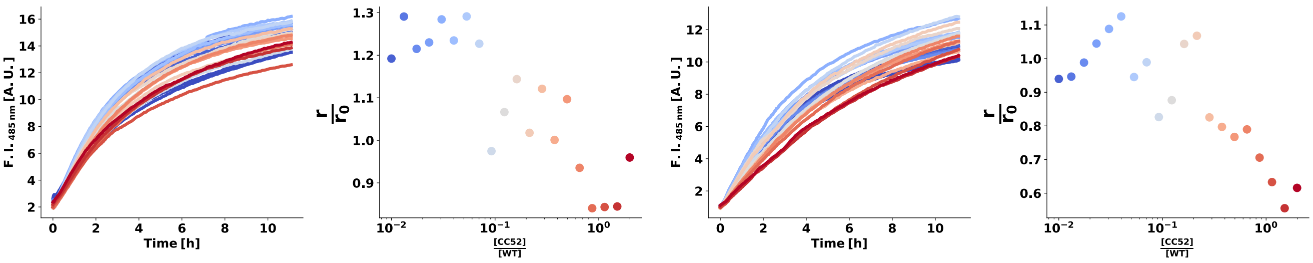

Fig. S5: CC52 dependency of WT fibril elongation. Two independent experiments Tht fluorescence over time of 25 µM WT monomer, 2.5 µM fibril seeds, with varying concentration of CC52, as well as the extracted relative elongation rates. Concentrations are plotted on a logarithmic x-axis, hence the 0 µM samples are not shown.

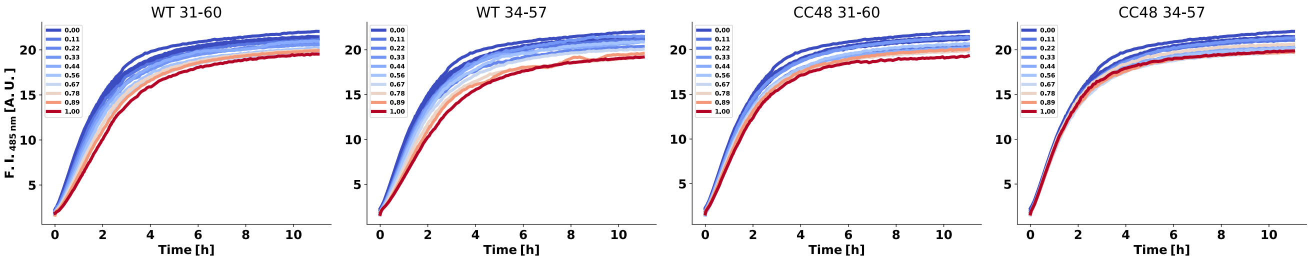

Fig. S6: Hairpin peptide dependency of WT fibril elongation. Tht fluorescence over time of 25 µM WT monomer with varying concentration of peptide, the sequence of which is indicated above the plot using the numbering from the parent protein. The legend shows the concentrations as the ratio between hairpin peptide and the WT monomer concentration.

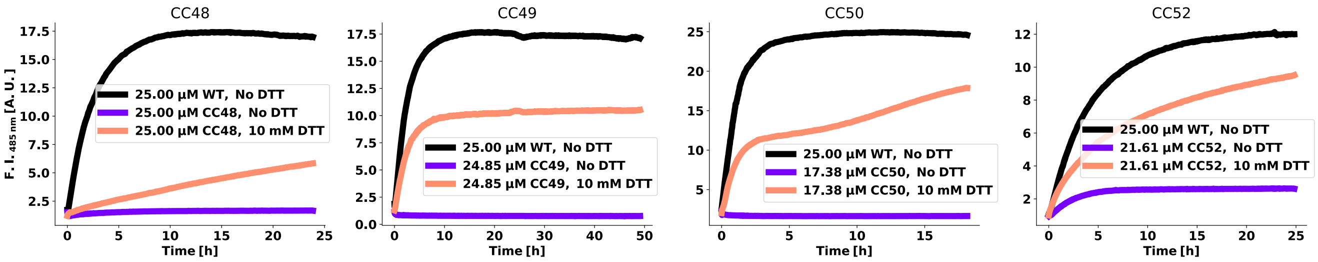

Fig. S7: DTT dependent incorporation of CC variants. Tht fluorescence over time of seeds in the presence of: WT monomer, CC variant, or CC variant with the reducing agent DTT. The specific CC variant is indicated above each plot, and the concentrations used are indicated in the legends.

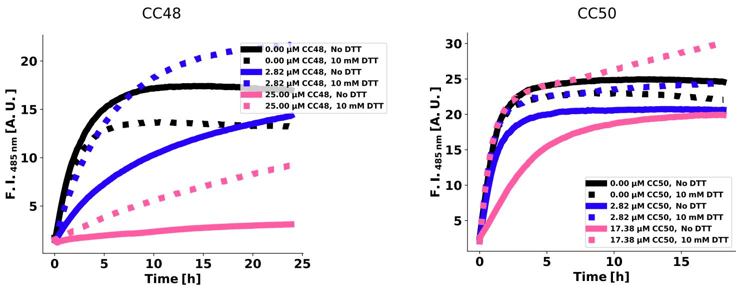

Fig. S8: DTT dependency of inhibition of WT fibril elongation. Tht fluorescence over time of WT filbril seeds in the presence of WT monomer and CC variant, either with or without the reducing agent DTT. The specific CC variant is indicated above each plot, and the concentrations used are indicated in the legends.

(a)

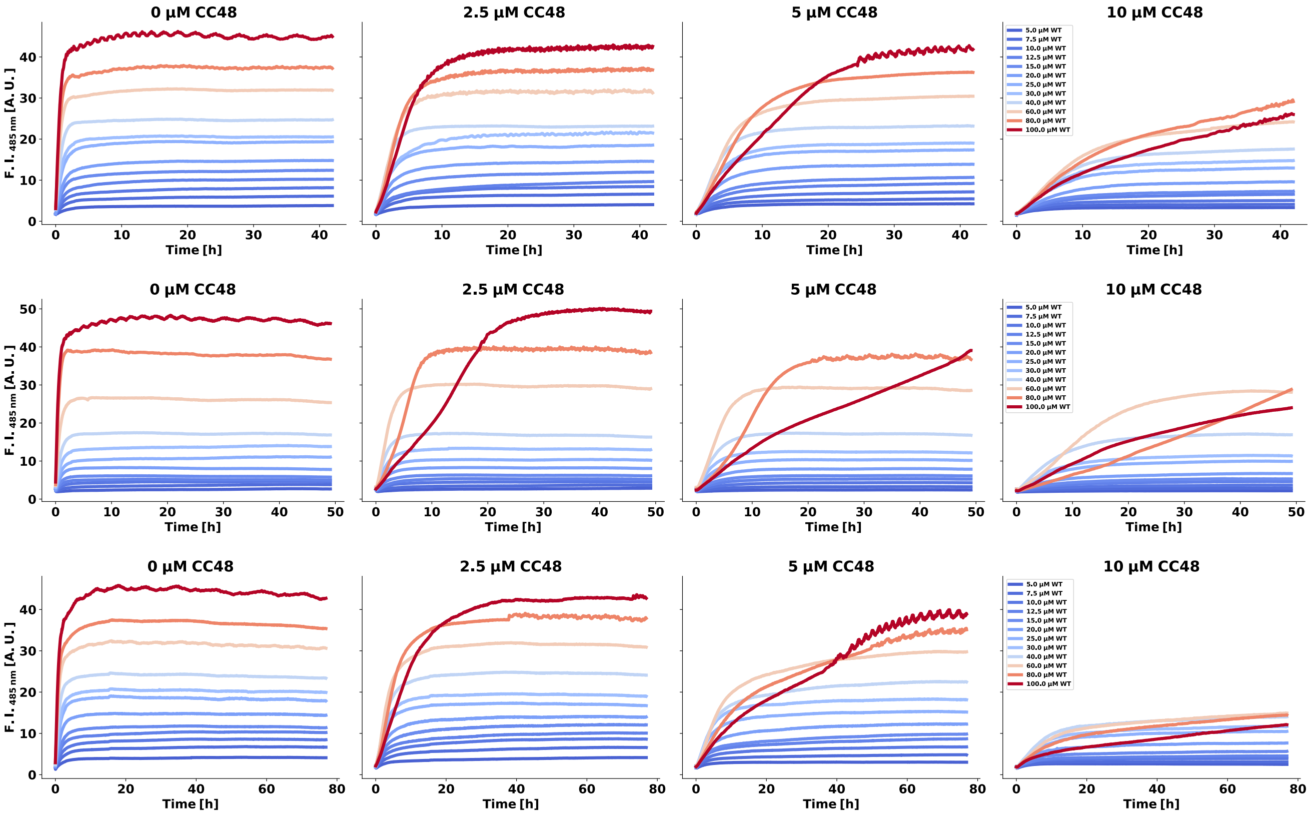

(b)

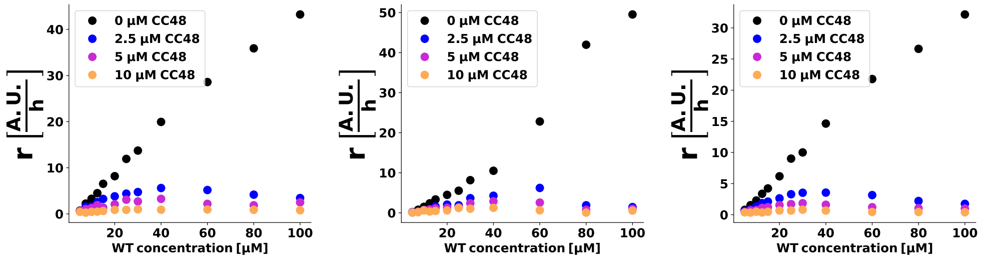

Fig. S9: WT monomer dependency of the inhibitory activity of CC48 on WT fibril elongation. (a) Three independent experiments of Tht fluorescence of 2.5 μM WT fibril seeds over time in the presence of fixed CC48 concentrations with a varying concentration of WT monomer indicated in the panels on the right. (b) The initial rates extracted from (a), note that this figure is shown in the main manuscript as well, shown here for completeness.

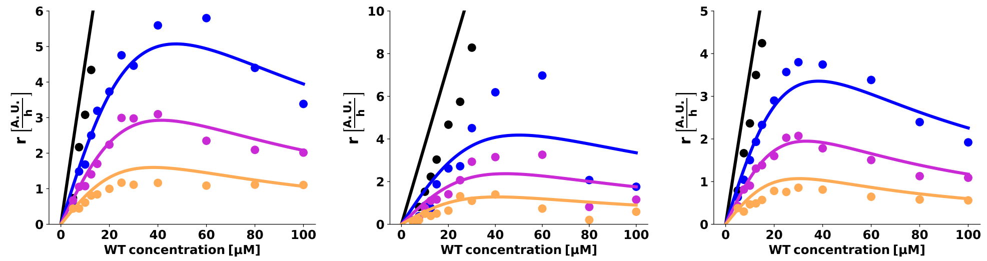

Fig. S10: Zoom-in and fit of the initial rates for the three independent experiments shown in Figure S9b. Note that left panel is also shown in the main manuscript.

Dataset #	K_m $[10^{-4}$ M]	K_i $[10^{-6}$ M]	K_1 $[10^{-1}$ M]	K_2 $[10^{-9}$ M]	$R_{\max}$ $[10^{-4}$ M]
1	5.5 ± 1.9	2.2 ± 0.41	$8.8 \pm 1.8 * 10^{-13}$	1.4 ± 0.37	2.6 ± 0.75
2	$2.1 * 10^2 \pm 5.3 * 10^5$	2.0 ± 2.43	$3.7 \pm 9.6 * 10^4$	$3.7 \pm 9.6 * 10^4$	$7800 \pm 200 * 10^5$
3	4.0 ± 0.86	2.4 ± 0.44	$9.2 \pm 2.3 * 10^{-14}$	0.82 ± 0.22	1.5 ± 0.27

Table S1: The parameters extracted from fitting using the package scipy.optimize with associated uncertainties in fitting. Note the large difference in the magnitude of parameters as well as the middle set being numerical very different from the other two sets.

3.1 Proxy initial slopes for dimeric constructs

For several of the dimeric constructs (see *e.g.* Figure S18, and Figure S20), a small kink in the initial growth phase was observed that became more prominent with increasing WT monomer concentration. Not only did it interfere with extraction of the initials slopes, it also raised the question if pure elongation was really what was seen in these experiments. It should be noted that the concentration of the dimeric constructs needed to induce inhibition was exceedingly low, in the nano molar range. Due to these low concentrations, it might take longer to establish the fibril-end-inhibitor equilibrium , giving rise to an initial burst growth phase. In order to test this, experiments were performed where the dimers and fibril-end were pre-incubated together before addition of WT (Figure S11). This pre-incubation led to disappearance of the initial kink. In order to get some "initial" slope, extraction of slopes were done after these kinks and compared to the initial slopes from the pre-incubation experiments performed at the same concentrations WT monomer, fibril seeds, and dimers (Figure S12). As the two kinds of slopes was in good agreement, the slopes extracted after the initial growth bursts was used as a proxy for initial slopes.

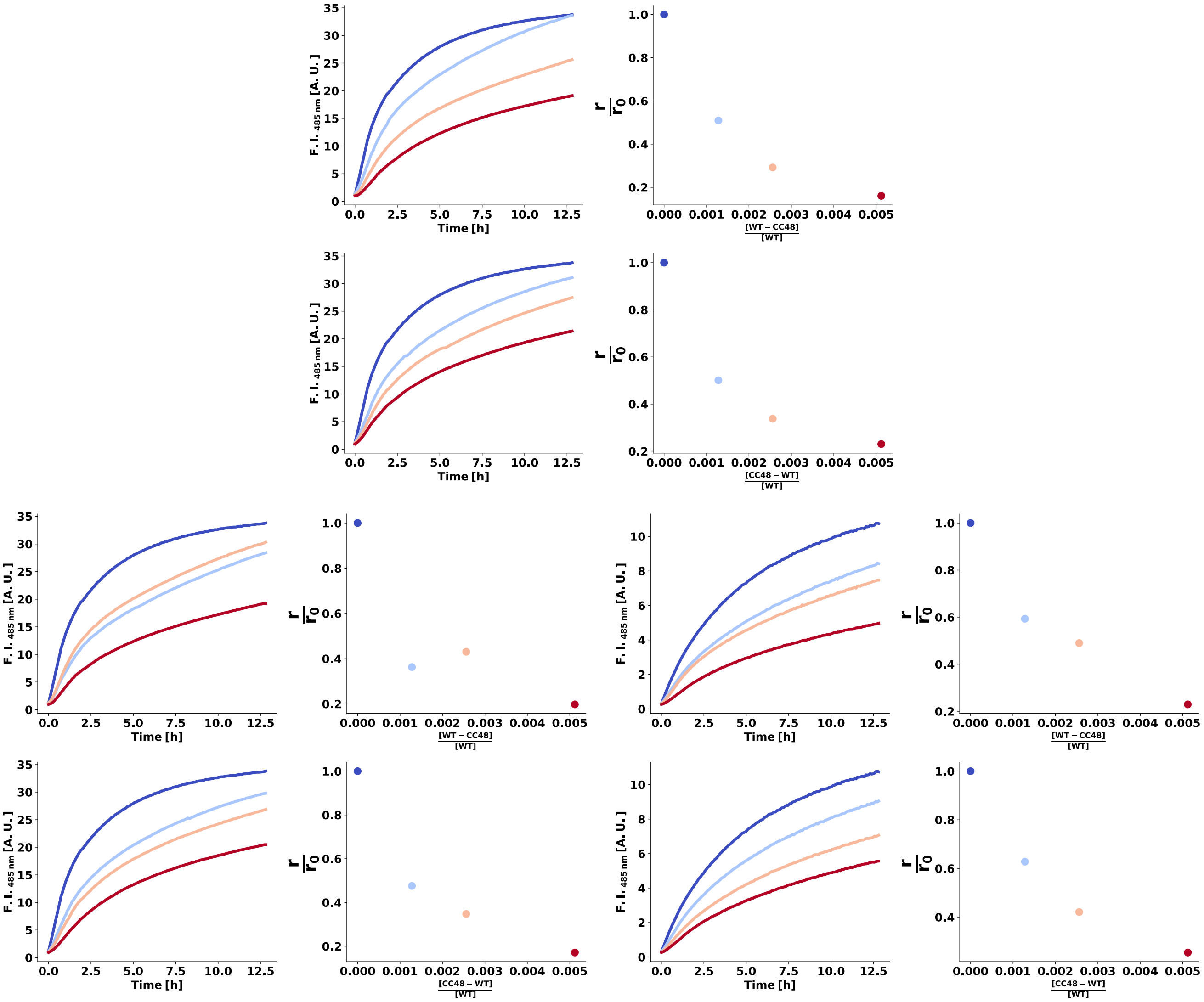

Fig. S11: Three independent experiments of Tht fluorescence over time and relative initial rates using a fixed concentration of 100 µM WT monomer that was mixed with 2.5 µM seeds that had been pre-incubated with different concentrations of WT-CC48 and CC48-WT dimers.

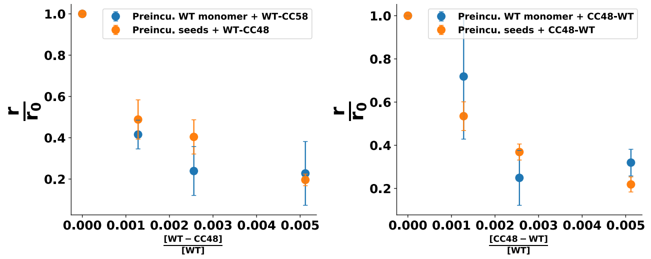

Fig. S12: Comparison between average initial rates from experiments where WT fibril seeds and inhibitor were pre-incubated (Figure S11), and where monomer and inhibitor were pre-incubated (Figure S19 and Figure S21).

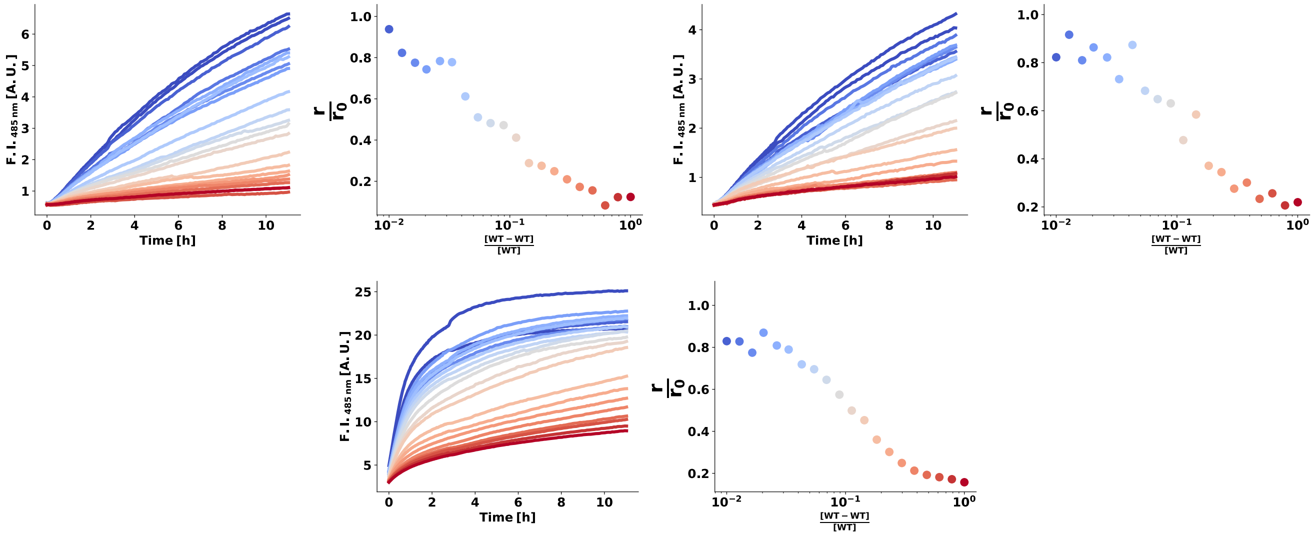

Fig. S13: WT-WT dependency of WT fibril elongation. Three independent experiments of Tht fluorescence over time of 25 µM WT monomer, 2.5 µM fibril seeds, with varying concentration of WT-WT, as well as the extracted relative elongation rates. Concentrations are plotted on a logarithmic x-axis, hence the 0 µM samples are not shown.

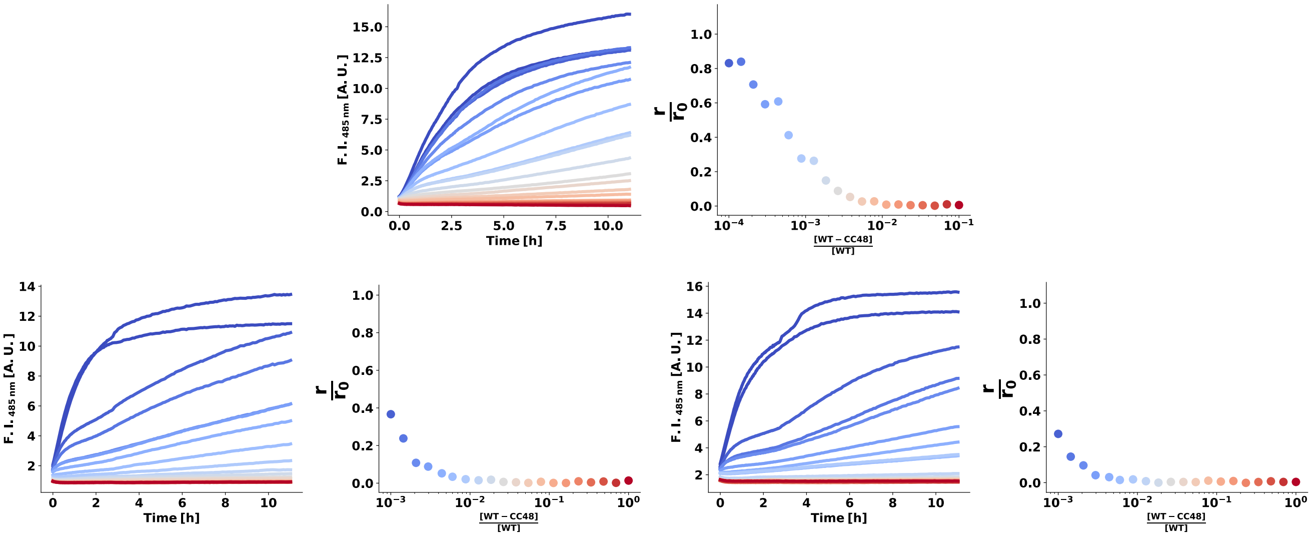

Fig. S14: WT-CC48 dependency of WT fibril elongation. Three independent experiments of Tht fluorescence over time of 25 µM WT monomer, 2.5 µM fibril seeds, with varying concentration of WT-CC48, as well as the extracted relative elongation rates. Concentrations are plotted on a logarithmic x-axis, hence the 0 µM samples are not shown. Note that different concentrations of WT-CC48 was used in the different experiments.

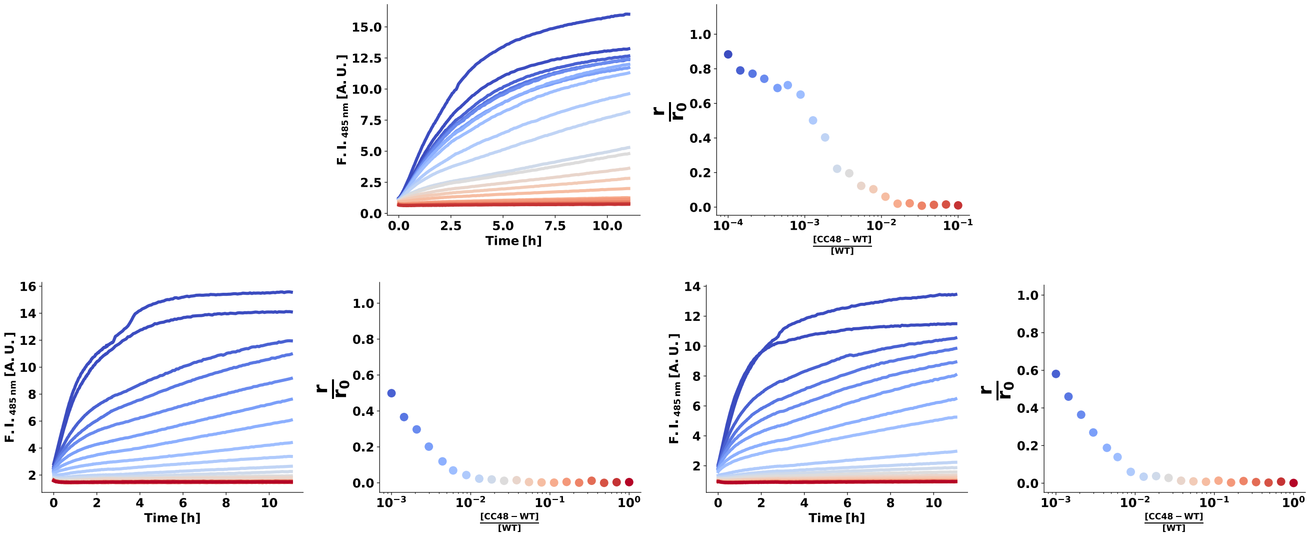

Fig. S15: CC48-WT dependency of WT fibril elongation. Three independent experiments of Tht fluorescence over time of 25 µM WT monomer, 2.5 µM fibril seeds, with varying concentration of CC48-WT, as well as the extracted relative elongation rates. Concentrations are plotted on a logarithmic x-axis, hence the 0 µM samples are not shown. Not that different concentrations of CC48-WT was used in the different experiments.

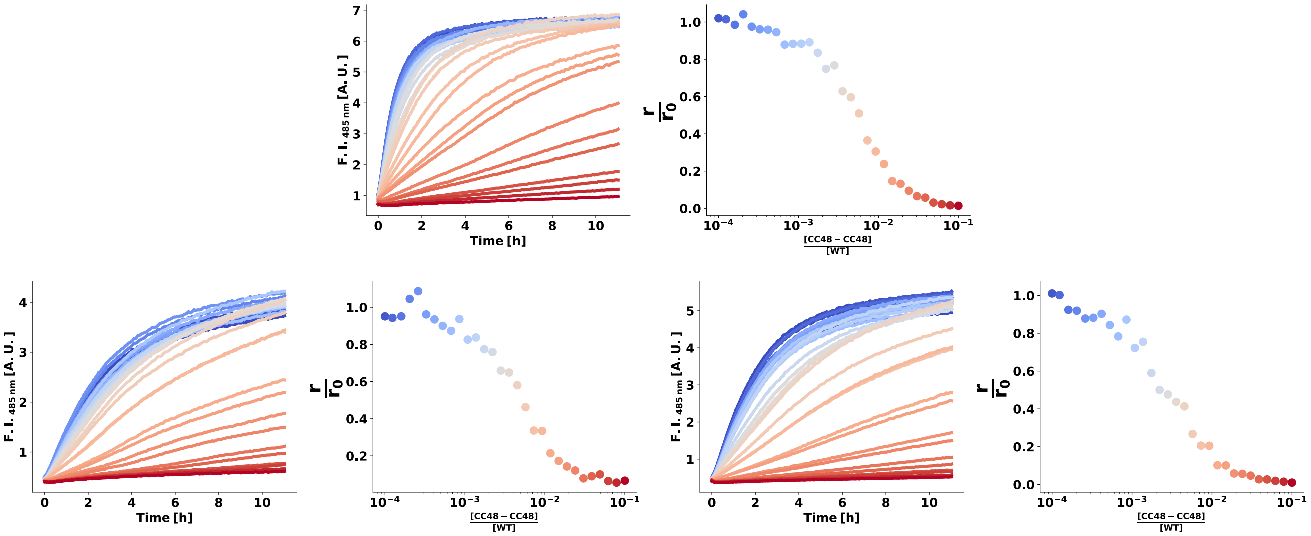

Fig. S16: CC48-CC48 dependency of WT fibril elongation. Three independent experiments of Tht fluorescence over time of 25 µM WT monomer, 2.5 µM fibril seeds, with varying concentration of CC48-CC48, as well as the extracted relative elongation rates. Concentrations are plotted on a logarithmic x-axis, hence the 0 µM samples are not shown.

(a)

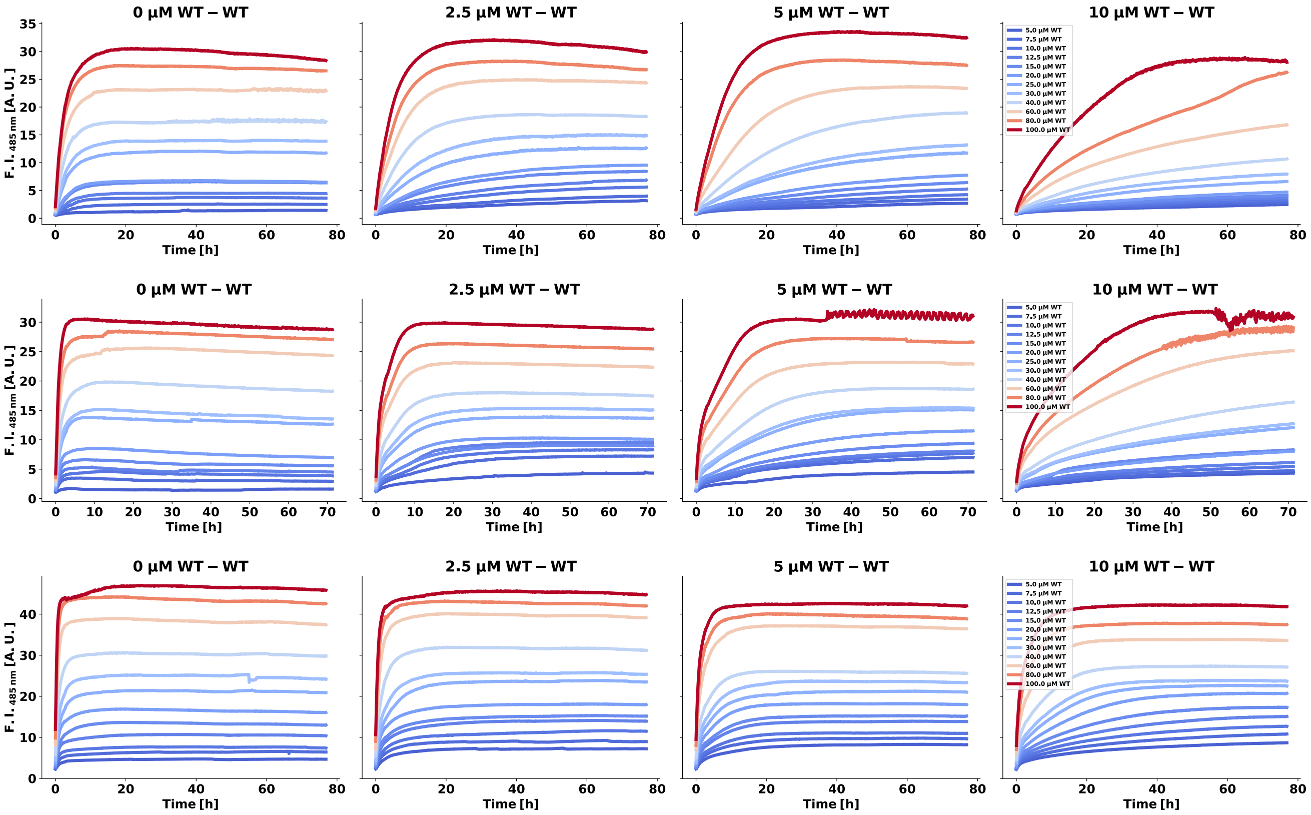

(b)

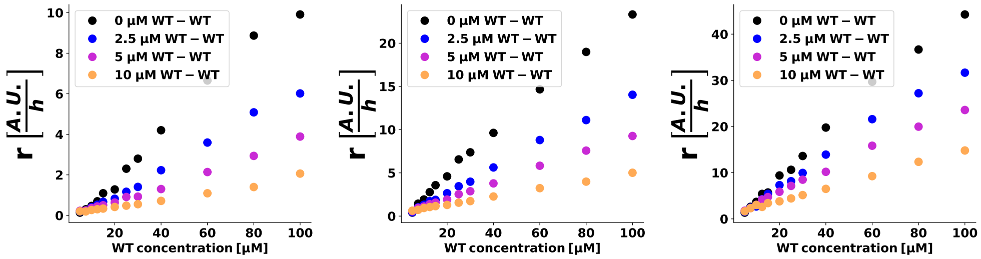

Fig. S17: WT monomer dependency of the inhibitory activity of WT-WT inhibition on WT fibril elongation. (a) Three independent experiments of Tht fluorescence of 2.5 µM WT fibril seeds over time in the presence of fixed WT-WT concentrations with varying WT monomer concentrations indicated in the rightmost panels. (b) The initial rates extracted from (a).

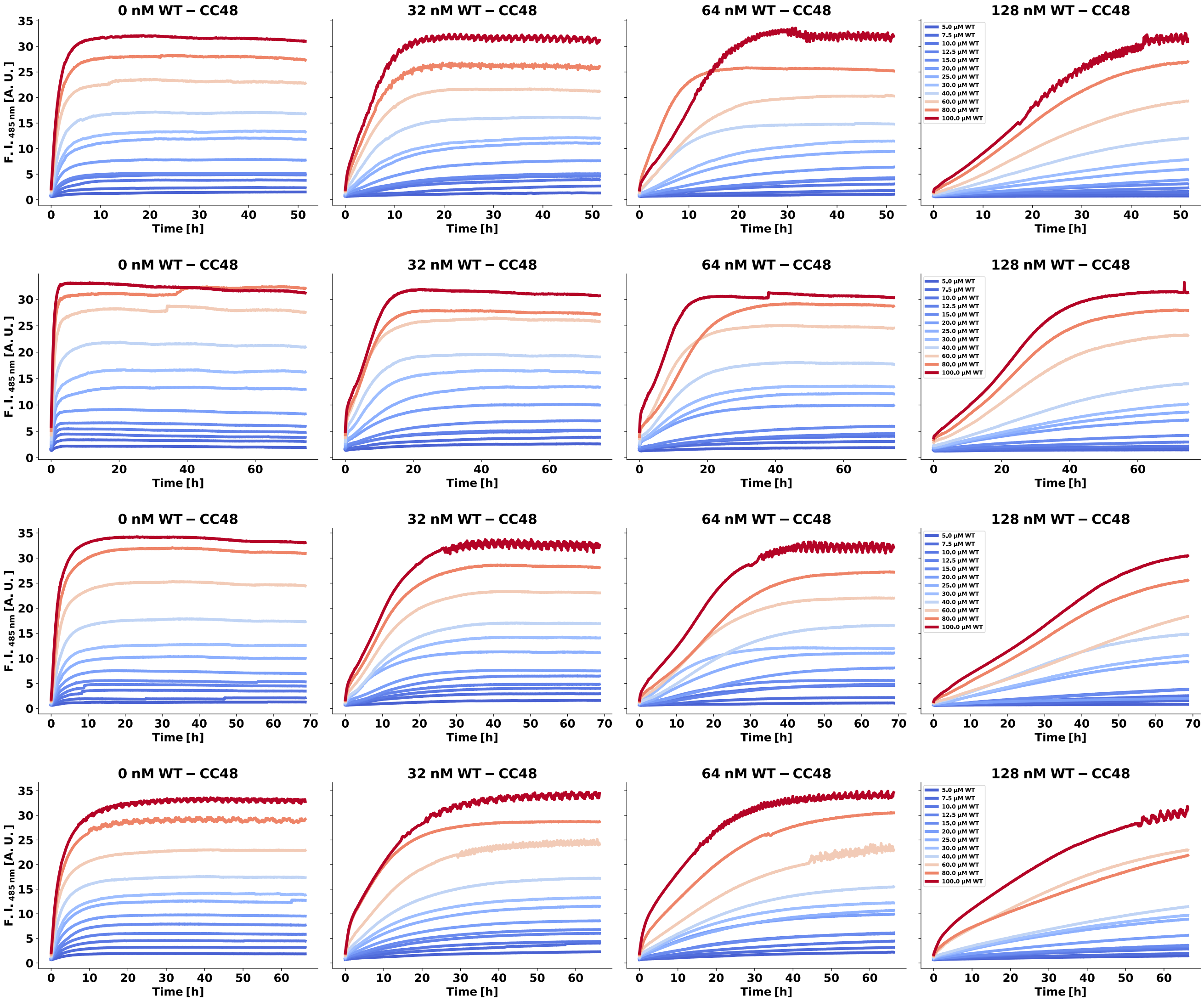

Fig. S18: WT monomer dependency of the inhibitory activity of WT-CC48 inhibition on WT fibril elongation. Four independent experiments of Tht fluorescence of 2.5 µM WT fibril seeds over time in the presence of fixed WT-CC48 concentrations with varying WT monomer concentrations indicated in the right-most panels.

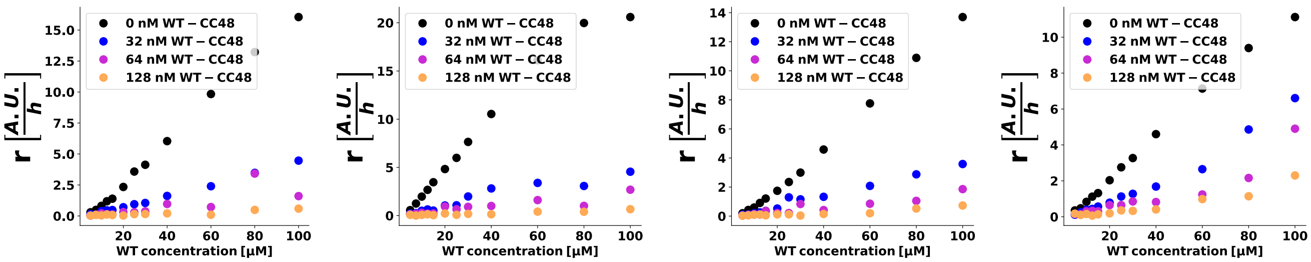

Fig. S19: The initial rates extracted from Figure S18.

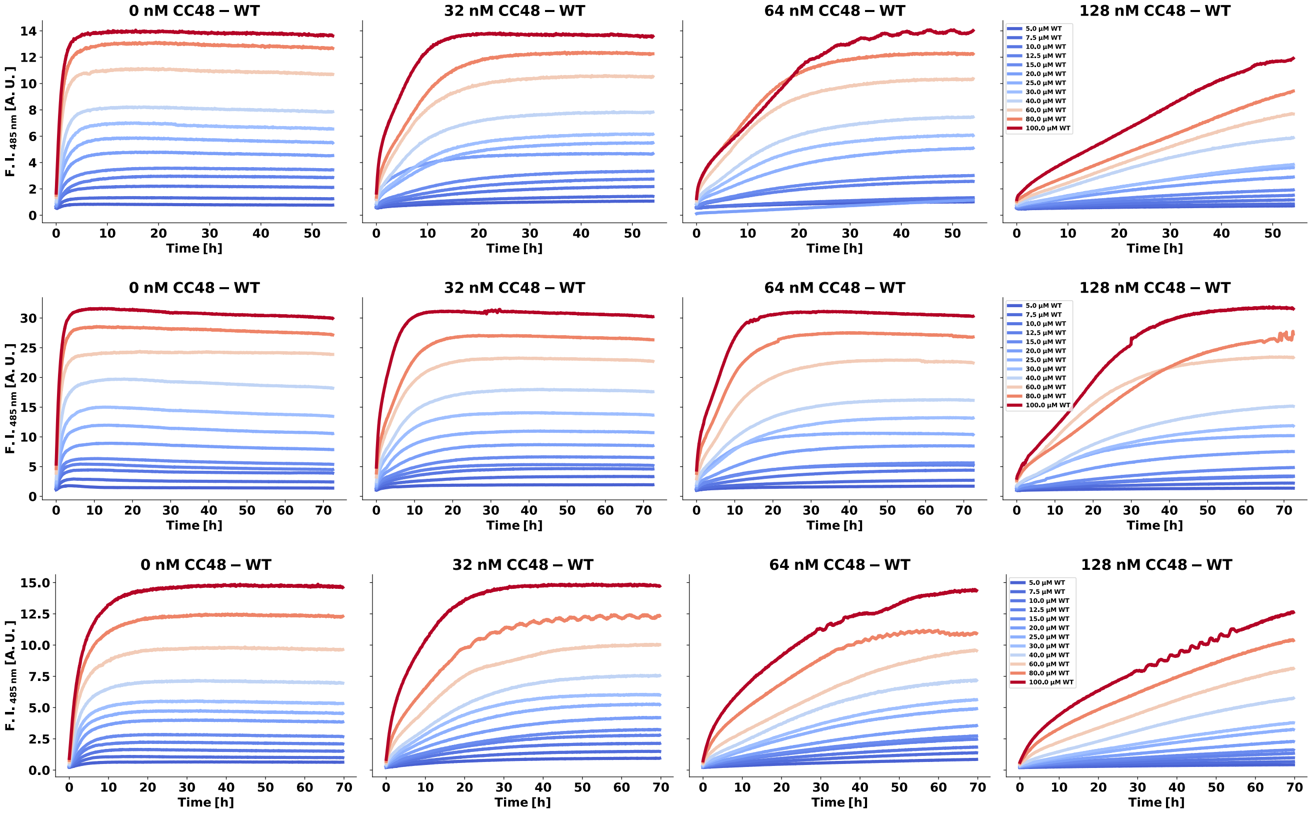

Fig. S20: WT Monomer dependency of the inhibitory activity of CC48-WT inhibition on WT fibril elongation. Three independent experiments of Tht fluorescence of 2.5 µM WT fibril seeds over time in the presence of fixed CC48-WT concentrations with varying WT monomer concentrations indicated in the right-most panels.

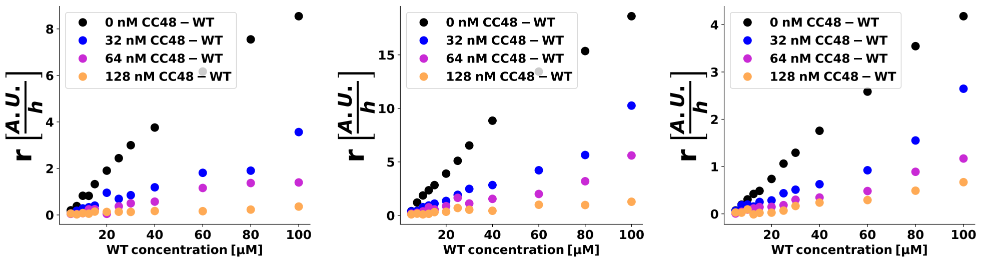

Fig. S21: The initial rates extracted from Figure S20.

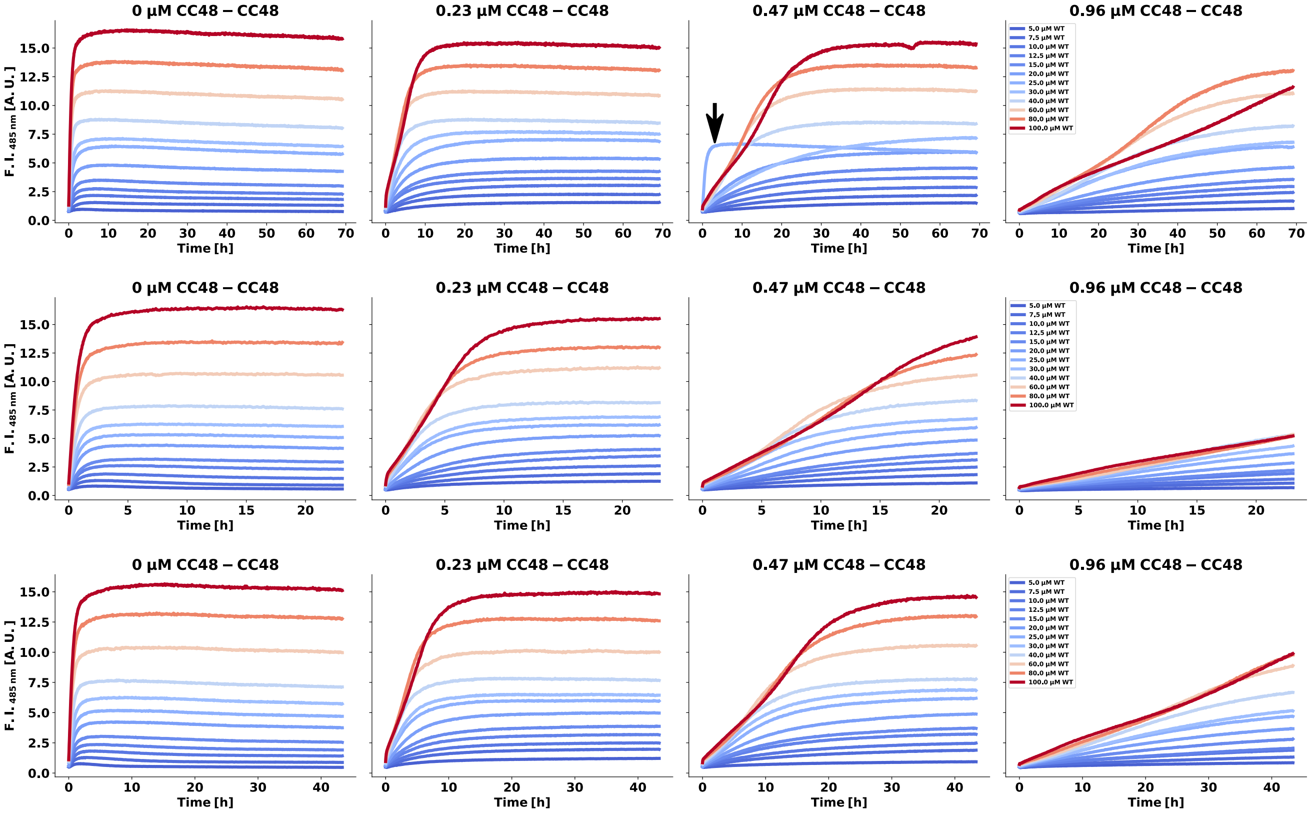

Fig. S22: WT Monomer dependency of the inhibitory activity of CC48-CC48 inhibition on WT fibril elongation. Three independent experiments of Tht fluorescence of 2.5 µM WT fibril seeds over time in the presence of fixed CC48-CC48 concentrations with varying WT monomer concentrations indicated in the rightmost panels.

(a)

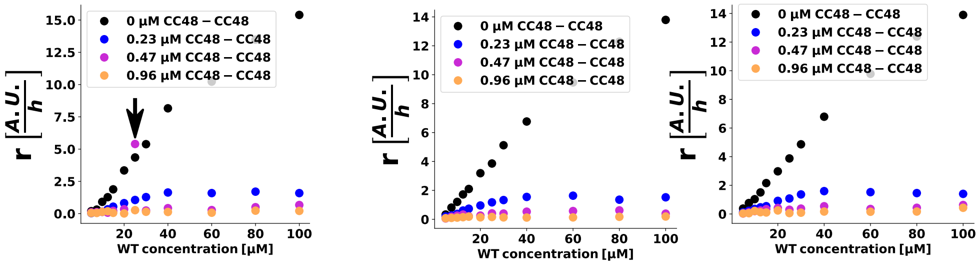

(b)

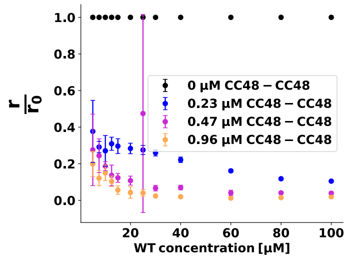

Fig. S23: WT Monomer dependency of the inhibitory activity of CC48-CC48 inhibition on WT fibril elongation continued. (a) The initial rates extracted from Figure S22, where the black arrows points to an outlier. (b) Relative and averaged initial slope of the rates in (a).

References

[1] H. Shaykhalishahi, A. Gauhar, M. M. Wördehoff, C. S. R. Grüning, A. N. Klein, O. Bannach, M. Stoldt, D. Willbold, T. Härd and W. Hoyer, *Angewandte Chemie International Edition*, 2015, **54**, 8837–8840.

[2] H. M. Beyer, P. Gonschorek, S. L. Samodelov, M. Meier, W. Weber and M. D. Zurbriggen, *PLOS ONE*, 2015, **10**, e0137652.

[3] M. Johnson, A. T. Coulton, M. A. Geeves and D. P. Mulvihill, *PLoS ONE*, 2010, **5**, e15801.

[4] W. Hoyer, T. Antony, D. Cherny, G. Heim, T. M. Jovin and V. Subramaniam, *Journal of Molecular Biology*, 2002, **322**, 383–393.

[5] M. Grey, S. Linse, H. Nilsson, P. Brundin and E. Sparr, *Journal of Parkinson's Disease*, 2011, **1**, 359–371.

[6] T. Scheibel, J. Bloom and S. L. Lindquist, *Proceedings of the National Academy of Sciences*, 2004, **101**, 2287–2292.

[7] A. K. Buell, J. R. Blundell, C. M. Dobson, M. E. Welland, E. M. Terentjev and T. P. J. Knowles, *Physical Review Letters*, 2010, **104**, 228101.

[8] K. Milto, A. Botyriute and V. Smirnovas, *PLoS ONE*, 2013, **8**, e68684.

[9] A. K. Buell, C. Galvagnion, R. Gaspar, E. Sparr, M. Vendruscolo, T. P. J. Knowles, S. Linse and C. M. Dobson, *Proceedings of the National Academy of Sciences*, 2014, **111**, 7671–7676.

[10] W. P. Esler, E. R. Stimson, J. M. Jennings, H. V. Vinters, J. R. Ghilardi, J. P. Lee, P. W. Mantyh and J. E. Maggio, *Biochemistry*, 2000, **39**, 6288–6295.

[11] A. J. Dear, G. Meisl, T. C. T. Michaels, M. R. Zimmermann, S. Linse and T. P. J. Knowles, *The Journal of Chemical Physics*, 2020, **152**, 045101.

[12] B. Li, P. Ge, K. A. Murray, P. Sheth, M. Zhang, G. Nair, M. R. Sawaya, W. S. Shin, D. R. Boyer, S. Ye, D. S. Eisenberg, Z. H. Zhou and L. Jiang, *Nature Communications*, 2018, **9**, 3609.

[13] Y. Li, C. Zhao, F. Luo, Z. Liu, X. Gui, Z. Luo, X. Zhang, D. Li, C. Liu and X. Li, *Cell Research*, 2018, **28**, 897–903.

[14] R. Guerrero-Ferreira, N. M. Taylor, D. Mona, P. Ringler, M. E. Lauer, R. Riek, M. Britschgi and H. Stahlberg, *eLife*, 2018, **7**, e36402.

[15] R. Guerrero-Ferreira, N. M. Taylor, A.-A. Arteni, P. Kumari, D. Mona, P. Ringler, M. Britschgi, M. E. Lauer, A. Makky, J. Verasdonck, R. Riek, R. Melki, B. H. Meier, A. Böckmann, L. Bousset and H. Stahlberg, *eLife*, 2019, **8**, e48907.

[16] X. Ni, R. P. McGlinchey, J. Jiang and J. C. Lee, *Journal of Molecular Biology*, 2019, **431**, 3913–3919.

[17] M. Schweighauser, Y. Shi, A. Tarutani, F. Kametani, A. G. Murzin, B. Ghetti, T. Matsubara, T. Tomita, T. Ando, K. Hasegawa, S. Murayama, M. Yoshida, M. Hasegawa, S. H. W. Scheres and M. Goedert, *Nature*, 2020.

[18] A. G. Marangoni, *Enzyme Kinetics*, John Wiley & Sons, Inc., Hoboken, NJ, USA, 2002.
